# Compassion and decision fatigue among healthcare workers during COVID-19 pandemic in a Colombian sample

**DOI:** 10.1371/journal.pone.0282949

**Published:** 2023-03-24

**Authors:** Gabriela Fernández-Miranda, Joan Urriago-Rayo, Verónica Akle, Efraín Noguera, Natalia Mejía, Santiago Amaya, William Jimenez-Leal

**Affiliations:** 1 Laboratory of Moral Judgments and Emotions, Universidad de los Andes, Bogotá, Colombia; 2 Psychology Department, Universidad de los Andes, Bogotá, Colombia; 3 Imagination and Modal Cognition Lab, Duke University, Durham, North Carolina, United States of America; 4 Neuroscience and Circadian Rhythms Laboratory, Universidad de los Andes, Bogotá, Colombia; 5 School of Medicine, Universidad de los Andes, Bogotá, Colombia; 6 Philosophy Department, Universidad de los Andes, Bogotá, Colombia; University of Klagenfurt, AUSTRIA

## Abstract

Being compassionate and empathic while making rational decisions is expected from healthcare workers across different contexts. But the daily challenges that these workers face, aggravated by the recent COVID-19 crisis, can give rise to compassion and decision fatigue, which affects not only their ability to meet these expectations but has a significant negative impact on their wellbeing. Hence, it is vital to identify factors associated to their exhaustion. Here, we sought to describe levels of compassion and decision fatigue during the pandemic, and to identify factors related to these forms of exhaustion. We collected data using self-reported questionnaires to measure compassion fatigue, decision fatigue, and grit in five intervals from April to November, 2020 (N = 856). Our results showed a negative correlation between grit and compassion and decision fatigue. We also found that under the circumstances studied grit tends to be higher in technicians, nurses, other professionals (psychologists, social workers), and workers at the Emergency Room (ER), and lower in general practitioners. Compassion fatigue tend to be higher for technicians, whereas decision fatigue was lower for specialists, general practitioners, and technicians, and higher for those working at private hospitals.

## Introduction

Some occupations and working contexts are more challenging than others. Roles that require empathizing with other people, particularly when they are suffering, may increase the demands of a job. That is why nurses, physicians, psychologists, psychiatrists, and educators, among others, experience a higher risk of emotional, cognitive, and physical exhaustion [1–7]. A clear example is the case of healthcare workers, who have a vital role in attending people experiencing health issues, which has become particularly evident in the COVID-19 pandemic. In this study, we explore the relationship between decision fatigue, compassion fatigue, and grit, a personality trait that has been related to the ability to overcome challenges [8,9]. We focused on a population of healthcare workers in Bogotá, Colombia during the initial period of the pandemic.

Figley [3] suggests that occupations that involve helping others who suffer require compassion, empathy, and an effort to take the perspective of the sufferer. However, this comes with a cost. People in caregiving occupations may experience tension and preoccupation as a result of being compassionate and empathic with their patients. The main feature of compassion fatigue is that it undermines the ability to experience empathy and compassion toward others and reduces caregivers’ capacity and interest in bearing other’s suffering [3,10–12]. Being compassionate with patients is one of the principles of medical ethics, so the deterioration of this ability has serious consequences in the healthcare practice [13].

Risk factors that predict compassion fatigue had been identified by several authors. Figley [3] asserted that the ongoing demand to relieve the sufferer’s pain, the prolonged exposure to it, the traumatic memories shared during the relationship, and the life disruptions in the caregiver’s personal life are predictors of compassion fatigue. Other authors added that its onset is related to organizational features such as high stress working environments, limited resources, poor quality of working relationships, and less experience in the job [4,11,14]. Newell and MacNeil [15] state that caregivers who experience compassion fatigue may have similar symptoms than those from Post-Traumatic Stress Disorder (PTSD), such as intrusive thoughts and traumatic memories related to their patients. They can also experience feelings of irritability and anger, sleeping disturbances, and difficulty when concentrating. Also, they may show avoidant behaviors and hypervigilant reactions. Compassion fatigue may result in reduced endurance, diminished performance, lack of energy, and a desire to quit [11,16].

Traditionally, emotional exhaustion has been evaluated under the construct of burnout. Burnout is defined as a psychological syndrome of emotional exhaustion, depersonalization, and reduced personal accomplishment that results from prolonged exposure to stressors at work [17]. Some of the features that distinguish compassion fatigue from burnout are that while the former results from specific exposure to events, the latter results from long-term involvement in emotionally demanding situations (not necessarily caregiving relationships). Compassion fatigue also has a faster symptomatology onset, is highly treatable, and has a fast recovery. Finally, compassion fatigue is characterized by a sense of helplessness and lack of social support [3]. Thus, we believe that the concept of compassion fatigue allows us to better capture some of the challenges presented by the pandemic because it results from the exposure to a particular event, the fast onset of the symptoms is presumably related to the viral outbreak, and most importantly, it gives information for developing treatment strategies.

As Dattilio argues, alongside compassion fatigue, professional challenges also affect healthcare workers’ cognitive resources and, therefore, the responses they provide to their patients. So, it is important in this regard to establish to what extent the fatigue experienced by healthcare workers extends from the emotional to a more cognitive domain. We evaluated this cognitive exhaustion using the construct of decision fatigue which refers to the impaired ability to make decisions and control behavior due to repeated acts of decision-making [18]. There is some evidence that suggests that decision fatigue affects surgeons [1], college students [19], and journal editors [20] and can be more widely understood as a manifestation of cognitive fatigue. However, is important to stress out that decision fatigue provides a theoretical framework to understand health workers experience, thus is not an established phenomenon.

Healthcare workers constantly have to make decisions regarding their patients’ treatments. Some of the behaviors used to characterize decision fatigue are avoidant behavior, procrastination tendencies, passivity, less persistence, and impulsivity. Additionally, it has been argued that decision fatigue deteriorates physical endurance, reduces executive functioning, inhibits reasoning ability, and results on the use of cognitive heuristics. Experiencing the features that have been understood as decision fatigue would affect healthcare workers’ choices, which have a crucial impact on patients’ health outcomes and, therefore, it is critical to prevent it [21].

One of the risk factors that predict decision fatigue is the complexity of the decisions and their consequences. Complex and consequential decisions have a higher impact on decision fatigue [22]. Situational factors such as the time of the day [23], making decisions before or after lunch [1], and sleep deprivation [24] may also impact the level of decision fatigue.

We believe compassion and decision fatigue are constructs that can help us understand part of the experience of healthcare workers in the context of the COVID 19 pandemic and beyond. Healthcare workers have been subjected to extended periods of time where their abilities to empathize and to make adequate decisions have been stressed and, as a result, might be impaired, likely resulting in deficient treatment for their patients. Hence, it is vital to identify factors related to decision and compassion fatigue in the healthcare system.

Figley [3] states that one of the protective factors against compassion fatigue is a sense of achievement and satisfaction with one’s effort. This is closely related to the concept of compassion satisfaction, which refers to the pleasure of helping others and making a difference in the world [25]. The ability to disengage and take distance from the professional setting also seems to be crucial to diminish fatigue [3]. This is accomplished by exerting self-care (exercising, having healthy eating habits, building supportive social networks, etc.) and being able to balance work and personal life [15,26]. Finally, cultivating resilience can also be a protective factor against compassion fatigue since it has been found a negative correlation between them [27]. To our knowledge, protective factors against decision fatigue are not clearly identified in the literature.

Grit is a personality trait that might constitute a protective factor for compassion and decision fatigue. It is understood as the tendency to pursue long-term goals with enthusiasm and hard work [28], and can predict success. This concept has two dimensions: perseverance in effort and consistency of interest. Both dimensions are related to being able to achieve long-term goals and work hard to overcome challenges even in absence of positive feedback [8,9]. According to a recent revision by Duckworth et al., these two factors are highly correlated and should not be treated as separate factors [29], a suggestion that we adopt in what follows.

Traditionally, most personality traits are considered to be stable during time. However, Duckworth states that it is possible to enhance grit and cultivate behaviors related to it [30]. Evidence from educational settings has shown that indeed grit can improve through different kinds of interventions [31–35]. For instance, an intervention carried out with medical students showed a significant change in grit levels that had a positive impact on their academic performance [36]. The potential to enhance grit through interventions as a protective factor for healthcare workers’ fatigue makes it pressing to dig deeper and understand how this phenomenon works within the healthcare system. Still, before moving on to interventions, it is important to document whether grit, decision fatigue, and compassion fatigue are related in ways consistent with such interventions.

In a recent concept analysis by Schimschal et al. [37], the authors identified several positive outcomes of grit. Not only are higher levels of grit related to improved levels of commitment, performance, retention, happiness, and well-being. Also, evidence suggests that it reduces burnout, attrition, anxiety, stress, and depression. If there is a negative correlation between grit and fatigue, this would open up the possibility of designing and testing interventions that aim to reduce fatigue by promoting grit in healthcare workers. Previous studies have explored the relationship between grit and fatigue in health workers. Doolittle [38] found that higher levels of grit were associated with lower levels of compassion fatigue and greater levels of compassion satisfaction. Other studies evaluated the relationship between grit and emotional exhaustion through the construct of burnout. These studies suggest an inverse relationship between grit and burnout in emergency medicine residents, surgery residents, gynecological residents, senior surgeons, and internal medical physicians [38–43]. Regarding cognitive exhaustion, to our knowledge, there are no studies that address the possible relationship between grit and decision fatigue, but it is a possibility worth exploring because cognitive fatigue impacts the treatment decisions that healthcare workers make and, hence, their patients’ wellbeing [1].

Besides the goals of intervention, research on the link between grit and fatigue can be useful to understand the experience of healthcare workers in the context of the pandemic. It is clear that pandemic outbreaks pose a great challenge to healthcare workers, who have a vital role in facing these crises. Their work might result in exhaustion and affect their occupational outcomes and, consequently, their patients. Recently, Sirois and Owens [44] published a systematic review of 138 empirical papers about factors associated with psychological distress during infectious disease outbreaks. The study was not limited to the COVID-19 pandemic, but most of the papers reviewed were carried out in this context. First, the authors identified that sociodemographic factors as age, sex, marital status, and educational level are potential risk or protective factors. Most of the studies showed that younger workers and females had higher risk of experiencing higher distress. Second, occupational role was also considered, and several studies showed that nurses were at higher risk of experiencing burnout and mental health issues. Third, social factors as interpersonal support, organizational support and clear communication were protective factors and buffered distress.

Only one of the 138 empirical papers that explore factors associated with distress in crises scenarios as the COVID-19 pandemic that were included in Sirois and Owens [44] review evaluated grit as a potential protective factor. That study was carried out by Huffman et al., [45] and showed that high levels of grit and resilience were protective factors to face the increase in the distress levels of medical providers, trainees, and administrators. This result suggests a relationship between grit and fatigue in health workers that should be observable during the COVID-19 pandemic.

The objective of the current study is to describe the levels of self-reported decision and compassion fatigue experienced by healthcare workers during the beginning of the COVID-19 pandemic and find how they are related to self-reported grit measures. We also intend to identify other sociodemographic factors that might influence decision and compassion fatigue, in line with the literature reviewed. If there is a negative correlation between grit and fatigue, as the literature suggest, this information offers a valuable input for development and testing of interventions useful for health workers during regular and critical times, especially in Middle Income countries that face a shortage of human resources [46].

## Method

### Participants

The sample included 856 healthcare workers from different hospitals in Bogotá (Colombia), working in person or remotely during the COVID-19 pandemic. Details of the sample are detailed in Table 1; briefly, 66.6% were women, 54.4% of them were over 40 years old, and the majority had a professional or a postgraduate degree (82.2%). The data were collected between April and November 2020, in five-time intervals (Apr 22-May 8; May 11-June 1; July 17-Aug 19; Sept 29-Nov 2; Nov 4-Nov 18) with varying numbers of observations (326, 147, 105, 85, and 193 participants, respectively).

**Table 1:** Sociodemographic characterization of the sample.

	Female	Male	Total
Position			
Specialists	186 (25.07)	149 (20.08)	335 (45.15)
General_practitioner	70 (9.43)	37 (4.99)	107 (14.42)
Resident	15 (2.02)	6 (0.81)	21 (2.83)
Nurse	63 (8.49)	16 (2.16)	79 (10.65)
Other_professional	75 (10.11)	20 (2.69)	95 (12.80)
Technician	73 (9.84)	13 (1.75)	86 (11.59)
Student	12 (1.62)	7 (0.94)	19 (2.56)
Total	494 (66.58)	248 (33.42)	742 (100)
Service area			
ER	96 (15.84)	53 (8.75)	149 (24.59)
ICU	56 (9.24)	44 (7.26)	100 (16.50)
Inpatient_unit	110 (18.15)	48 (7.92)	158 (26.07)
Outpatient_unit	134 (22.11)	42 (6.93)	176 (29.04)
Private_practice	4 (0.66)	10 (1.65)	14 (2.31)
Telehealth	4 (0.66)	5 (0.83)	9 (1.49)
Total	404 (66.66)	202 (33.34)	606 (100)
Hospital type			
Private	312 (41.8)	158 (21.2)	470 (63)
Public	182 (24.4)	94 (12.6)	276 (37)
Total	494 (66.2)	252 (33.8)	746 (100)

The table shows the number and percentage (in parenthesis) of participants by Service area, Position, and Hospital type grouped by gender. The difference in the total n is due to lost data from sociodemographic information.

Participants were contacted using email addresses publicly available on the websites of the four Health Services Networks in Bogotá, Colombia. Invitations were also sent through institutional emails in two hospitals. The invitation email included the link to the Qualtrics survey. In the end, participants had the option to include their email if they were interested in participating in the following measurements. For each new measurement period, invitations were sent to “old” and new participants. Since participation was anonymous, participants didn’t provide data that allowed for identification across measurement periods.

### Instruments

This study used four self-report questionnaires to measure decision fatigue, compassion fatigue, grit, and some specific behaviors that can show grit in the healthcare context. We chose the most used and widely accepted questionnaires to measure these constructs. To reduce the time required to respond to the questionnaires we selected the items with the higher factor loadings in Duckworth & Quinn [9] Hickman et al., [47] and Adams et al., [12] and removed the others (see items removed below and S1 File.) Additionally, we consulted experts in the healthcare system to validate that removing those items was appropriate considering the context of the participants. Each questionnaire was translated into Spanish by two bilingual speakers in the areas of health care and social research. Only afterwards, the questionnaires with the selected items were validated in a pilot sample of 10 people to validate comprehension and time of questionnaire completion before running the study.

#### Grit

Health workers’ level of grit was assessed using the Short Grit Scale (Grit-S) [9]. The Grit-S consists of two factors: perseverance in effort (4 items) and consistency in interest (4 items). In each factor, the item with the lowest factorial load was removed: *I am a hard worker* and *I have difficulty maintaining my focus on projects that take more than a few months to complete*. This way, the study used a 6-item assessment (three for each factor), each rated on a 5-point Likert-type scale from 1 (*Not like me at all*) to 5 (*Very much like me*). The items in the interest consistency factor were reversed to get consistent scores. Observed reliability for the sample was α = 0.7 and ω = 0.77 (more information in S1 File). The two factors were averaged into a single score for the data analysis considering the recently acknowledged lack of evidence for the two-factor structure.

#### Decision fatigue

This construct was measured through the Decision Fatigue Scale (DFS) [47]. The DFS is a unidimensional 10-item measure. However, for this study the three items with the lowest factor load reported in original validation studies were removed: *someone else should make decisions for me*, *I have made decisions quickly in order to move on* and *I have made decisions without carefully thinking about them*. Therefore, the study used a 7-item version of the scale, each rated on a 4-point Likert-type scale from 0 (*strongly disagree*) to 3 (*strongly agree*). Observed reliability for the sample was α = 0.88 and ω = 0.88 (More information in S1 File). Notice that although decision fatigue is defined as the impaired ability to make decisions and control behavior due to repeated acts of decision making, the items of the scale focus on the difficulty to make decisions, but not on its cause. However, this was the best validated measure to assess this construct.

#### Compassion fatigue

Participants’ level of compassion fatigue was assessed using the Compassion Fatigue Short Scale (CF-Short Scale) [12]. The scale consists of 13 items divided into two factors: an 8-item job burnout subscale and a 5-item secondary stress subscale. In each factor, the item with the lowest factor load was eliminated: *I have thoughts that I am not succeeding in achieving my life goals* and *I have experienced intrusive thoughts after working with an especially difficult client/patient*. Thus, this study used an 11-item assessment, each scored on a linear numeric scale from 1 (*rarely/never*) to 10 (*very often*). Observed reliability for the sample was α = 0.88 and ω = 0.89 (More information in S1 File).

We used an additional instrument to evaluate grit behaviors in the healthcare context, however, the observed reliability for the sample was α = 0.61 and ω = 0.68, so it was not included in the data analysis (more information in S1 File).

### Procedure

A web survey was conducted in Spanish on the Qualtrics platform. After the written informed consent was read and accepted, the instruments were presented in a random order to ensure that none were predictably affected by a fatigue effect. At the end of the survey, participants responded to a set of questions on general demographic characteristics and specific features related to the healthcare context. An open-response question was also included in which participants could express their feelings and personal experience regarding the COVID-19 pandemic. This was included, following a suggestion by officials and medical directors of the hospitals that collaborated with us, as an exploratory question–answers were not taken into account in the analyses. The median time of survey completion was 9 min. The study was approved by the Ethics Committee at Universidad de los Andes (Approval # 1168, 2020).

### Data analysis

Participants who completed less than 90% of the survey (388 observations) were excluded. After the exclusions, our final data set contained 856 observations. We performed some basic quality checks (range and value check). Then we ran the analysis including and excluding outliers, and there were no differences in the main results. Therefore, we did not eliminate any outliers, neither univariate nor multivariate, since we had no reason to believe they were not due to natural variation.

Analyses were conducted using the R statistical language [48]. First, a reliability analysis was performed to determine whether it was possible to create indices by averaging the scores assigned to items of each instrument and their subscales. Since data collection began prior to what would become the first peak of the pandemic, we decided to create a variable to capture the change between the moments before (from April 22 to July 21), during (from July 22 to August 19) and after (from September 29 to November 18) this first peak. Although data was collected in five moments, this was only for convenience and was done, of course, without knowledge of the ebb and flow of the pandemic in the city.

We first carried out an ANOVA test to examine whether there were differences between the three moments (pre-peak, peak, post-peak) regarding the variables of interest (grit, decision fatigue, compassion fatigue). We also explored the relationship between the COVID-19 cases in Bogotá, and the trajectory of our key variables. In order to identify relationships between the variables of interest and the sociodemographic variables, we fitted Generalized Estimating Equation-based Linear Models (GEELMs). GEELMs allow the estimation of population averages of covariates directly, as opposed to also estimating individual effects, as in mixed models, is less intensive computationally, has fewer assumptions, and reflects more adequately the observation clustering present in this study (for further information on GLEEMs see Agresti et al., [49] on advantages over mixed models see McNeish et al., [50] and Muth et al., [51]). For all models, missing data was deleted listwise, which explains small differences in sample sizes and degrees of freedom. Notice that we report raw descriptives for all variables (see Table 2), but all variables were standardized for model fitting and other inferential statistics.

**Table 2:** Overall descriptive statistics for the key variables of the study.

Time	Cases	Grit	Decision Fatigue	Compassion Fatigue
Pre	473	3.32 (0.68)—[1.33–4.5]	0.8 (0.73)—[0––3]	2.97 (1.78)—[1–8.63]
Peak	105	3.33 (0.74)—[1.16–4.5]	0.87 (0.77)—[0––3]	3.33 (1.82)—[1–9.18]
Post	278	3.5 (0.7)—[0.83–4.5]	0.64 (0.69)—[0––3]	2.49 (1.66)—[1––10]
Total	856	3.38 (0.7)—[0.83–4.5]	0.76 (0.73)—[0––3]	2.86 (1.76)—[1––10]

Note: Mean (SD)–[min value–max value]. Grit was rated on a 5-point Likert-type scale from 1 (Not like me at all) to 5 (Very much like me); Decision fatigue was rated on a 4-point Likert-type scale from 0 (strongly disagree) to 3 (strongly agree); Compassion fatigue was rated on a 10-point Likert-type scale from 1 (rarely/never) to 10 (very often). Ratings when then standardized using z-scores for the analysis.

## Results

Descriptive statistics are presented in Table 2. Overall, participants reported relatively low levels of compassion and decision fatigue (both means closer to the lower end of the range of scale) while levels of Grit were around the midpoint of the scale. Notice, however, that participants did not report high levels of Grit either (between 4.5 and 7, the highest possible value) and, with a small standard deviation, they tend to converge around the mean. This pattern holds for the three moments considered, with the largest apparent differences between the peak and post-peak means of all variables.

### Grit, decision fatigue, and compassion fatigue change across time

We conducted ANOVAs and post hoc tests (Tukey Correction) to examine overall differences between the time points for the key variables. There were significant differences in grit (*F*(2, 853) = 6.50, *p* < 0.01, η^2^ = 0.015), decision fatigue (*F*(2, 853) = 5.99, *p* < 0.01, η^2^ = 0.014), and compassion fatigue (*F*(2, 853) = 10.5, *p* < 0.001, *η*^2^ = 0.024) between collection points. Grit showed a tendency to increase over time while decision and compassion fatigue decreased over the same time period. However, compassion fatigue increased during the first peak the of pandemic in Bogotá, Colombia indicated by post-hoc tests. During this period, the mean number of daily cases in the country was of 8608 [52], and the mean number of daily cases in Bogotá was of 3300 [53]. This suggests that crises as the COVID-19 pandemic could be related to changes in the healthcare workers’ levels of compassion fatigue.

Given previous results, we performed additional analyses considering the pattern of the COVID-19 number of cases in Colombia during the data collection. We evaluated whether variance significantly differed between the three moments (pre-peak, peak, post-peak) using Interclass Correlation Coefficients (ICC) and Levene’s tests. These descriptive tools allow us to test for uniformity along another important data dimension and validate the need for GLEEMs. We found that the scores for compassion fatigue were significantly different for ICC (*ICC1* = 0.04, *F*(472,946), *p* < 0.05) but not for the Levene’s test. Decision fatigue also had significantly different variances in the three moments only according to Levene’s test (*F*(2) = 3.39, *p* < 0.05) but not to de ICC. This suggests that the peak of the pandemic is associated with larger variances for these key variables during the peak of the pandemic but not for grit, for which no significant differences were found. Bear in mind that the peak of the pandemic has the smallest number of observations (pre *=* 473, peak *=* 105, post *=* 278) which might explain these differences in variance. However, even in the face of these unequal samples per moment, Grit does not vary unlike the other variables which suggest there might be something else driving these differences.

### Predictors of compassion and decision fatigue

**Fig 1:**
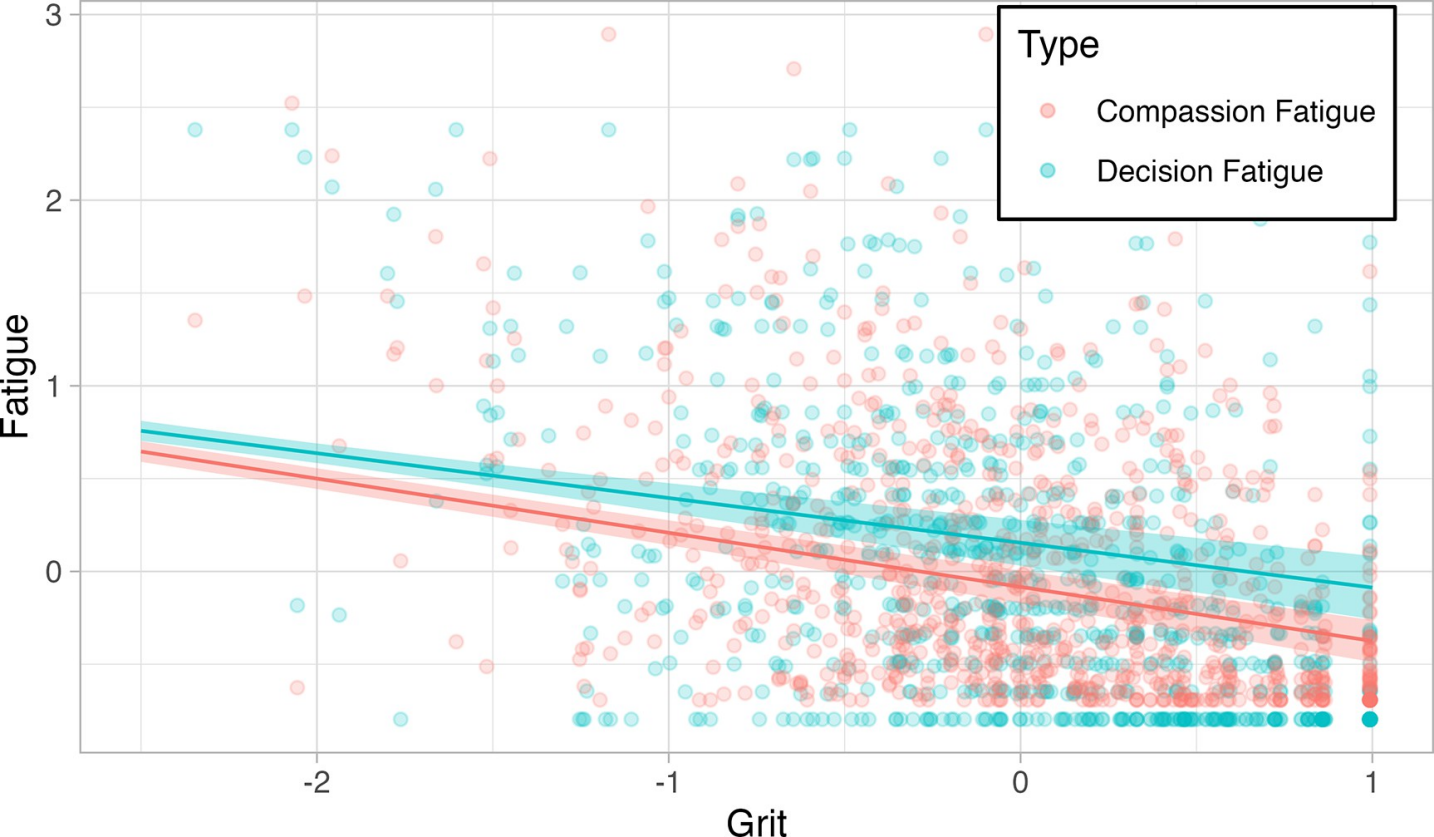
Inverse relation between grit and compassion fatigue & grit and decision fatigue. Lines show means and shaded areas 95% confidence intervals.

We fitted a series of GEELMs with Grit as the predictor variable and compassion and decision fatigue as outcomes, controlling for sociodemographic variables and testing for interactions between these and our key variables. Initially, we included time as a predictor as well, but since the results were not significant it was not included in subsequent models. The models were realized with the data of the 578 participants that reported all their sociodemographic information. Model selection was determined by using a Quasi Information Criterion (QIC) and the resulting model is presented in Table 3. We then carried out the subgroup analysis using Estimated Marginal Means with False Discovery Rate (FDR) adjustment. The GEELMs showed a significant inverse relationship between grit and decision fatigue (*B* = -0.24, *p* < 0.01), and between grit and compassion fatigue (*B* = -0.29, *p* < 0.01) (Fig 1). In other words, people with higher levels of grit tend to experience lower levels of compassion and decision fatigue.

**Table 3:** Generalized estimated equations-based linear models estimates.

	Grit	Decision fatigue	Compassion fatigue
Grit		-0.24***	-0.21***
		[-.29,-.19]	[-.32, -.26]
Decision fatigue	-0.19***		0.39***
	[-.26, -.12]		[.34, .43]
Compassion fatigue	-0.33***	0.56***	
	[-.35, -.30]	[.52,.59]	
Position			
General_practitioner	-0.16***	-0.03*	-0.02
	[-.17, -.14]	[-.06, .0]	[-.05, .01]
Nurse	0.09***	-0.10	0.04
	[.06, .11]	[-.30, .10]	[-.03, .11]
Other_professional	0.12***	0.07**	-0.10*
	[.09, .15]	[.02, 0.12]	[-.18, -.01]
Resident	-0.25***	0.22**	-0.05
	[-.27, -.23]	[.08, .36]	[-.13, .04]
Specialist	0.01	-0.11***	0.05
	[-.14, .14]	[-.17, .06]	[-.01, .10]
Student	0.04	0.15	-0.03
	[-.16, .25]	[-.09, .38]	[-.19, .13]
Technician	0.16***	-0.19***	0.10**
	[.11, .21]	[-.23, -.15]	[.08, .12]
Area			
ER	0.08**	-.11	0.07
	[.03, .12]	[-.24, .01]	[-.02, .16]
ICU	0.04	-0.08***	0.01
	[-.20, .28]	[-.12, -.04]	[-.04, .06]
Impatient_unit	0.08*	-0.05	-0.01
	[.00, .16]	[-.17, .07]	[-.10, .09]
Outpatient_unit	0.0	-0.07	0.02
	[-.08, .07]	[-.21, .08]	[-.04, .09]
Private_practice	-0.32**	0.37***	-0.34***
	[-.50, -.13]	[.28, .45]	[-.43, -.25]
Telehealth	0.12	-0.05	0.24
	[.01, .23]	[-.18, .08]	[.08, 0.4]
Intercept	-0.07***	0.15*	-0.08
	[-.11, -.04]	[.03, .27]	[-.17, .01]
N	578	578	578
Pseudo R^2^	0.31	0.40	0.439
QIC	189.2	224.5	159.0

*** p < .01

** p < .05

* p < .1. Numbers in brackets are confidence the intervals for each coefficient. Complete Table of the model including Hospital type, Gender, and Age in S1 Table.

**Fig 2:**
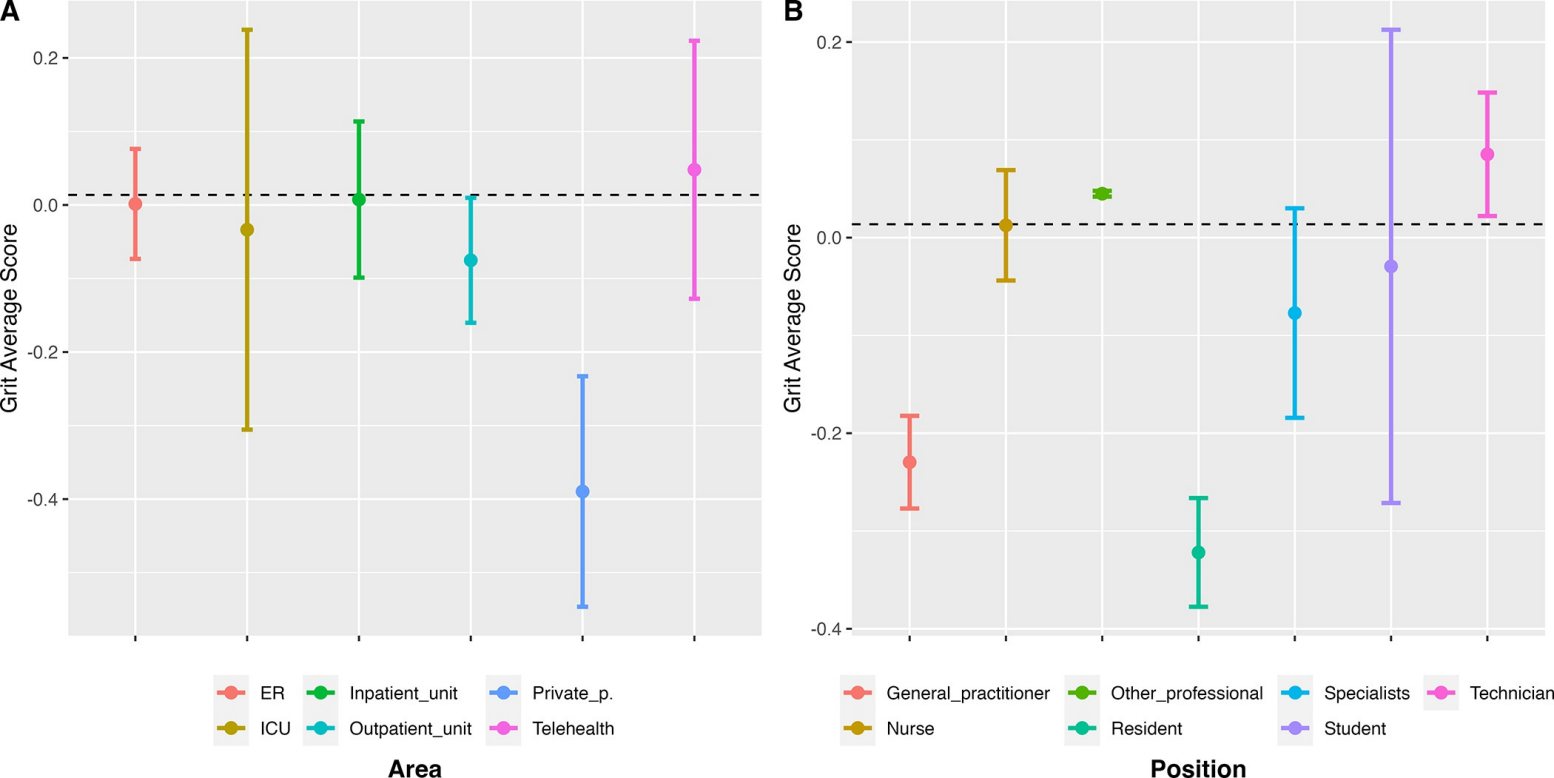
Grit ratings by service area and position. (A) Service area vs. grit & (B) Position vs. grit.

We also found that certain service areas or positions are associated with different grit levels (Fig 2). On the one hand, some positions and service areas are associated with being grittier than the general mean. This is the case of nurses (*B* = 0.09, *p* < 0.01), technicians (*B* = 0.16, *p* < 0.01) and other healthcare professionals (psychologists, social workers, etc.) (*B* = 0.12, *p* < 0.01), who report significantly higher levels of grit. Participants who work on the emergency room (ER) also report having significantly higher levels of grit (*B* = 0.08, *p* < 0.01). Additionally, participants who had between 26 and 30 years reported significantly higher levels of grit (*B* = 0.10, *p* < 0.01).

On the other hand, there are positions and service areas associated with being less gritty. First, general practitioners and residents reported significant lower levels of grit, *B* = -0.16, *p* < 0.01 and *B* = -0.25, *p* < 0.01, respectively. Second, people who work in private practice also reported significantly lower levels of grit (*B* = -0.32, *p* < 0.01) (see Fig 2). However, since the sample size for residents (n = 21) and private practice (n = 14) was small, and the variability is high, these results are not generalizable.

**Fig 3:**
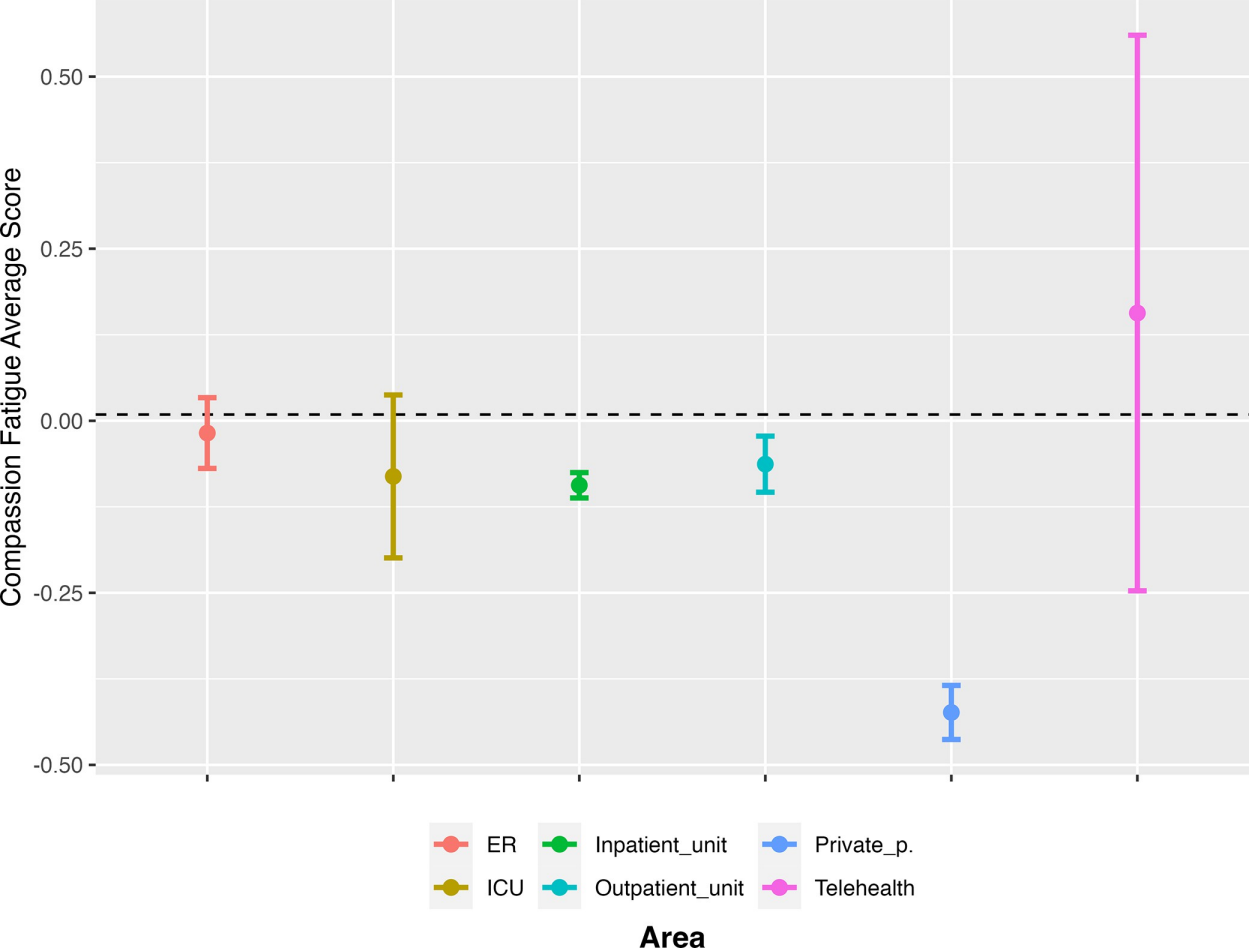
Compassion fatigue ratings by service area.

We also found some factors that are associated with experiencing compassion fatigue. For instance, participants working in private practice (*B* = -0.34, *p* < 0.01) reported significantly lower levels of compassion fatigue (Fig 3). However, the sample size for this area is small and does not allow to generalize this finding. On the contrary, technicians reported significantly higher levels of compassion fatigue (*B* = 0.10, *p* = 0.01). More generally, it seems that age and gender are associated with compassion fatigue. We found that participants who were between 26 and 30 years old (*B* = 0.10, *p* < 0.01) and between 31 and 35 years old (*B* = 0.11, *p* < 0.01) reported experiencing significantly higher levels of compassion fatigue. Lastly, female participants reported significantly higher levels of compassion fatigue (*B* = 0.11, *p* < 0.01).

Decision fatigue is also associated with certain positions and service areas. Participants who worked as specialist physicians (*B* = -0.11, *p* < 0.01), general practitioners (*B* = -0.03, *p* < 0.01), technicians (*B* = -0.19, *p* < 0.001), and those working in the ICU (*B* = -0.08, *p* < 0.001) reported significantly lower levels of decision fatigue. On the contrary, residents (*B* = 0.22, *p* < 0.001), other professionals (*B* = 0.07, *p* < 0.05), participants working in private practice (*B* = 0.37, *p* < 0.001), and those working in private hospitals (*B* = 0.02, *p* < 0.05) reported higher levels of decision fatigue, albeit small sample sizes for these groups prevent us from making a strong inference.

Given the differences between different positions and service areas on compassion and decision fatigue we also evaluated the presence of interactions between these. We found a small effect for some service areas so that at low levels of compassion fatigue grit levels are high, but at high levels of compassion fatigue, grit vary for residents, technicians, other professionals, and workers in the ICU. These small statistical effects prevent us from drawing strong conclusions on the possible moderating factor of holding these positions but suggest an intriguing pattern that deserves to be looked at independently (see S1 Fig for further information about the interactions).

## Discussion

Our study shows an inverse relation between grit and fatigue. In other words, grittier people tend to experience less compassion fatigue and less decision fatigue. Below, we proceed to present the results for each of the key constructs in our study.

### Compassion fatigue

Overall, we found a tendency to decrease over time in the compassion fatigue scores. However, there was a significant increase in this variable during the first peak of the pandemic in Bogotá, Colombia. This led us to perform further analyses that showed more variance in compassion fatigue during the peak of the pandemic compared to the lapse pre-peak and post-peak.

The fact that compassion fatigue scores increased during the peak of COVID-19 cases and that the variance was larger at this point supports Jenkins and Warren [11] thesis that high-stress working environments are risk factors for compassion fatigue. It also suggests that, as Figley [3] states, the ongoing demand to relieve the sufferer’s pain may result in compassion fatigue. However, this temporal association between the levels of compassion fatigue and the COVID-19 number of cases increase is also evidence of the faster onset and faster recovery of compassion fatigue compared to burnout [3]. Therefore, compassion fatigue may be an accurate way to identify and measure emotional exhaustion changes in healthcare workers during crises such as the COVID-19 pandemic.

Previous literature showed that certain positions in the healthcare system represented a risk factor to experience distress [44]. Particularly, some studies show evidence that nurses were at higher risk of experiencing psychological distress [54]. In our study, we didn’t find significant results for this position, but we did find that technicians had higher levels of compassion fatigue. Additionally, as Sirois and Owens [44], we found that age and gender are potential risk factors for emotional exhaustion evaluated as compassion fatigue. In our study, female participants and those between the ages of 26 and 35 reported higher levels of compassion fatigue.

### Decision fatigue

As it was the case of compassion fatigue, we found a tendency to decrease over time for the decision fatigue scores. Likewise, variance in the scores was larger during the peak of the pandemic. These results suggest that decision fatigue is malleable across time as well. We also identified differences in decision fatigue across positions and service areas. Specialists, general practitioners, and technicians reported lower levels of decision fatigue while those working in private hospitals reported higher levels.

Considering decision fatigue in the context of healthcare workers is important because they are constantly in the position of making choices that affect the patients’ health. For instance, previous studies have found evidence that orthopedic surgeons tend to authorize fewer surgeries after continued sessions of making decisions (e.g., before lunch and at the end of their shift) [1]. Also, it has been found evidence of decision fatigue in the case of judges in parole hearings who are more likely to give parole when the hearings take place early in the morning than in the afternoon [55]. In both, the surgeons’ and the judges’ cases, it seems that decision fatigue results in them taking the default–safest–option.

A possible explanation for the low levels of reported decision fatigue in our study may be that healthcare workers have appropriate training to make decisions in the context of crisis as the COVID-19 pandemic. The result can also be related to specific protocols of the hospitals that make it easier for the workers to make choices about the patient’s treatment. This may explain the difference between those working in public hospitals and those working in private hospitals (who report experiencing higher levels of decision fatigue). However, it is not enough to rely on self-reports to arrive at these conclusions and further studies must be carried out. Finally, the Decision Fatigue Scale does not fully capture the construct, which is a limitation of the present study.

### Grit

We found a negative correlation between grit and compassion and decision fatigue, which is consistent with Huffman et al., [45] who suggests that grit can be a protective factor against exhaustion. This is also related to the fact that it has been argued that grit predicts success, endurance, high performance, high levels of energy, improved commitment, retention, and well-being [8,11,16,37]. Further work is necessary to establish whether grit is indeed protective factor of fatigue.

Interestingly, the inverse relationship between grit and compassion fatigue wasn’t consistent for age. Younger participants have high levels of grit, but also high levels of compassion fatigue, so in their case, it is necessary to explore possible interactions in future studies. We also found that general practitioners reported lower levels of grit while technicians, other professionals, and those working in the ER reported higher levels.

## Future directions

Our findings provide a valuable understanding of the experience of healthcare workers during the COVID-19 pandemic. However, further work is necessary to answer some questions raised by these results. First, we found some significant and interesting correlations for residents (lower levels of grit, higher levels of decision fatigue) and those working in private practice (lower levels of grit, lower levels of compassion fatigue, higher levels of decision fatigue). Further studies should explore the directionality of the effect between grit and fatigue. Future studies measuring fatigue and grit levels that target specific service areas and positions within the health system might be useful to obtain a more fine-grained information on how health crisis can differentially affect workers in the system. Second, we used self-report measures to evaluate compassion fatigue, decision fatigue, and grit. This implies that we are relying on participants’ perceptions of their internal states, and this sometimes may not be accurate. Particularly, in the case of decision fatigue, it would be useful to compare self-report measures with behavioral measures to evaluate to what extent they are experiencing this type of fatigue. Although studies with behavioral measures are hard to do in circumstances of real crises (time and resource availability in the health system), validations of this sort will be necessary prior to designing interventions with real effects in these environments. Third, a longitudinal within-subject study would be interesting to evaluate the changes across time that we suggest in our study.

## Conclusions

In conclusion, the results of our study provide us with a framework that allows a better understanding of the experience of health workers during the first part of the COVID-19 pandemic. Interestingly, compassion and decision fatigue were inversely correlated to reported levels of grit. These results are significant for, at least, two reasons. Our study is (to our knowledge) the first to provide evidence of inverse relations between grit and fatigue in situations of generalized crisis and uncertainty. These relations need to be better understood to develop and test interventions on factors associated to fatigue that health workers experience in situations of crisis. Intervening in those factors is crucial to promote well-being on health workers and their patients. Lastly, the fact that these effects might be differentially observed depending on service area and position paints a more nuanced picture of health workers experience of the pandemic, a picture where the effects of rising cases did not have uniformly effects on those who were working hard to save our lives in time of maximum uncertainty.

## Supporting information

S1 Fig Interaction results. (DOCX)

S1 Table Comparison results with full data, data without repeated participants, and data without outliers. (DOCX)

S1 File Full instruments and translations. (DOCX)

## References

[1] Persson E, Barrafrem K, Meunier A, Tinghög G. The effect of decision fatigue on surgeons’ clinical decision making. Health Econ. 2019 Oct;28(10):1194–203. doi: 10.1002/hec.3933 31344303 PMC6851887

[2] McCormack HM, MacIntyre TE, O’Shea D, Herring MP, Campbell MJ. The Prevalence and Cause(s) of Burnout Among Applied Psychologists: A Systematic Review. Front Psychol. 2018 Oct 16;9:1897. doi: 10.3389/fpsyg.2018.01897 30386275 PMC6198075

[3] Figley CR. Compassion fatigue: Psychotherapists’ chronic lack of self care. J Clin Psychol. 2002 Nov;58(11):1433–41. doi: 10.1002/jclp.10090 12412153

[4] Yoder EA. Compassion fatigue in nurses. Appl Nurs Res. 2010 Nov;23(4):191–7. doi: 10.1016/j.apnr.2008.09.003 21035028

[5] Koenig A, Rodger S, Specht J. Educator Burnout and Compassion Fatigue: A Pilot Study. Can J Sch Psychol. 2018 Dec;33(4):259–78.

[6] Smart D, English A, James J, Wilson M, Daratha KB, Childers B, et al. Compassion fatigue and satisfaction: A cross-sectional survey among US healthcare workers: Compassion Satisfaction and Burnout. Nurs Health Sci. 2014 Mar;16(1):3–10. 23663318 10.1111/nhs.12068

[7] Showalter SE. Compassion Fatigue: What Is It? Why Does It Matter? Recognizing the Symptoms, Acknowledging the Impact, Developing the Tools to Prevent Compassion Fatigue, and Strengthen the Professional Already Suffering From the Effects. Am J Hosp Palliat Med. 2010 Jun;27(4):239–42. doi: 10.1177/1049909109354096 20075423

[8] Duckworth AL, Peterson C, Matthews MD, Kelly DR. Grit: Perseverance and passion for long-term goals. J Pers Soc Psychol. 2007;92(6):1087–101. doi: 10.1037/0022-3514.92.6.1087 17547490

[9] Duckworth AL, Quinn PD. Development and Validation of the Short Grit Scale (Grit–S). J Pers Assess. 2009 Feb 17;91(2):166–74. doi: 10.1080/00223890802634290 19205937

[10] Figley CR. Compassion Fatigue as Secondary Traumatic Stress Disorder: An Overview. In: Compassion fatigue Coping with secondary traumatic stress disorder in those who treat the traumatized. New York: Brunner Routledge; 1995. p. 1–20.

[11] Jenkins B, Warren NA. Concept Analysis: Compassion Fatigue and Effects Upon Critical Care Nurses. Crit Care Nurs Q. 2012 Oct;35(4):388–95. doi: 10.1097/CNQ.0b013e318268fe09 22948373

[12] Adams RE, Boscarino JA, Figley CR. Compassion fatigue and psychological distress among social workers: A validation study. Am J Orthopsychiatry. 2006 Jan;76(1):103–8. doi: 10.1037/0002-9432.76.1.103 16569133 PMC2699394

[13] American Medical Association. Code of Medical Ethics: Patient-physician relationships [Internet]. Code of Medical Ethics overview. [cited 2022 Aug 8]. Available from: https://www.ama-assn.org/delivering-care/ethics/code-medical-ethics-patient-physician-relationships.

[14] Potter P, Deshields T, Divanbeigi J, Berger J, Cipriano D, Norris L, et al. Compassion Fatigue and Burnout: Prevalence Among Oncology Nurses. Clin J Oncol Nurs. 2010 Oct 1;14(5):E56–62. doi: 10.1188/10.CJON.E56-E62 20880809

[15] Newell JM, MacNeil GA. Professional Burnout, Vicarious Trauma, Secondary Traumatic Stress, and Compassion Fatigue: A Review of Theoretical Terms, Risk Factors, and Preventive Methods for Clinicians and Researchers. 13.

[16] Coetzee SK, Klopper HC. Compassion fatigue within nursing practice: A concept analysis: Concept analysis of compassion fatigue. Nurs Health Sci. 2010 Apr 19;12(2):235–43. 20602697 10.1111/j.1442-2018.2010.00526.x

[17] Maslach Christina, Jackson Susan E., Leiter Michael P. Maslach Burnout Inventory: Third edition. In: Zalaquett C. P., Wood R. J., editors. Evaluating stress: A book of resources. Scarecrow Education; 1997. p. 191–218.

[18] Dattilio FM. The Self‐Care of Psychologists and Mental Health Professionals: A Review and Practitioner Guide. Aust Psychol. 2015 Dec 1;50(6):393–9.

[19] Vohs KD, Baumeister RF, Schmeichel BJ, Twenge JM, Nelson NM, Tice DM. Making Choices Impairs Subsequent Self-Control: A Limited-Resource Account of Decision Making, Self-Regulation, and Active Initiative. J Pers Soc Psychol. 2008;94(5):883–98. doi: 10.1037/0022-3514.94.5.883 18444745

[20] Stewart AF, Ferriero DM, Josephson SA, Lowenstein DH, Messing RO, Oksenberg JR, et al. Fighting decision fatigue. Ann Neurol. 2012 Jan;71(1):A5–15. doi: 10.1002/ana.23531 22275264

[21] Pignatiello GA, Martin RJ, Hickman RL. Decision fatigue: A conceptual analysis. J Health Psychol. 2020 Jan;25(1):123–35. doi: 10.1177/1359105318763510 29569950 PMC6119549

[22] Oto B. When Thinking is Hard: Managing Decision Fatigue [Internet]. Emergency & Mobile Medicine Learning Network. 2012 [cited 2022 Aug 8]. Available from: https://www.hmpgloballearningnetwork.com/site/emsworld/article/10687160/when-thinking-hard-managing-decision-fatigue.

[23] Chan MY, Cohen H, Spiegel BMR. Fewer Polyps Detected by Colonoscopy as the Day Progresses at a Veteran’s Administration Teaching Hospital. Clin Gastroenterol Hepatol. 2009 Nov;7(11):1217–23. doi: 10.1016/j.cgh.2009.07.013 19631284

[24] Baldwin DC, Daugherty SR. Sleep Deprivation and Fatigue in Residency Training: Results of a National Survey of First- and Second-Year Residents. Sleep. 2004 Mar;27(2):217–23. doi: 10.1093/sleep/27.2.217 15124713

[25] Stamm BH. Helping the Helpers: Compassion Satisfaction and Compassion Fatigue in Self-Care, Management, and Policy of Suicide Prevention Hotlines. Resour Community Suicide Prev. 2012;1–4.

[26] Houck D. Helping Nurses Cope With Grief and Compassion Fatigue: An Educational Intervention. Clin J Oncol Nurs. 2014 Aug 1;18(4):454–8. doi: 10.1188/14.CJON.454-458 25095300

[27] Burnett HJ. The Compassion Fatigue and Resilience Connection: A Survey of Resilience, Compassion Fatigue, Burnout, and Compassion Satisfaction among Trauma Responders. Int J Emerg Ment Health Hum Resil [Internet]. 2015 [cited 2022 Aug 8];17(1). Available from: https://www.omicsonline.com/open-access/the-compassion-fatigue-and-resilience-connection-a-survey-of-resilience-compassion-fatigue-burnout-and-compassion-satisfaction-among-trauma-responders-1522-4821-17-165.php?aid=37926.

[28] Von Culin KR, Tsukayama E, Duckworth AL. Unpacking grit: Motivational correlates of perseverance and passion for long-term goals. J Posit Psychol. 2014 Jul 4;9(4):306–12. doi: 10.1080/17439760.2014.898320 31404261 PMC6688745

[29] Duckworth AL, Quinn PD, Tsukayama E. Revisiting the Factor Structure of Grit: A Commentary on Duckworth and Quinn (2009). J Pers Assess. 2021 Sep 3;103(5):573–5. doi: 10.1080/00223891.2021.1942022 34254861

[30] Duckworth AL. Grit: The Power of Passion and Perseverance. New York, London, Toronto, Sydney, New Delhi: SCRIBNER; 2016.

[31] Park D, Tsukayama E, Yu A, Duckworth AL. The development of grit and growth mindset during adolescence. J Exp Child Psychol. 2020 Oct;198:104889. doi: 10.1016/j.jecp.2020.104889 32629233 PMC8747892

[32] Major C. Youth Mentoring Partnership’s Friend Fitness Program: Theoretical Foundations and Promising Preliminary Findings from a New Positive Psychology Intervention for Grit and Positive Youth Development [Internet] [Master’s Theses]. [Pennsylvania]: University of Pennsylvania; 2013 [cited 2022 Aug 8]. Available from: https://repository.upenn.edu/mapp_capstone/48/.

[33] Sundar S, Qureshi A, Galiatsatos P. A Positive Psychology Intervention in a Hindu Community: The Pilot Study of the Hero Lab Curriculum. J Relig Health. 2016 Dec;55(6):2189–98. doi: 10.1007/s10943-016-0289-5 27460673

[34] Rhodes J, May J, Andrade J, Kavanagh D. Enhancing Grit Through Functional Imagery Training in Professional Soccer. Sport Psychol. 2018 Sep 1;32(3):220–5.

[35] Rusadi RM, Sugara GS, Isti’adah FN. Effect of mindfulness-based cognitive therapy on academic grit among university student. Curr Psychol [Internet]. 2021 May 6 [cited 2022 Aug 8]; Available from: https://link.springer.com/10.1007/s12144-021-01795-4.

[36] Mirza TI, Yasmeen R, Mahboob U. Nurturing Grit among Medical Students. Pak J Med Sci [Internet]. 2021 Feb 4 [cited 2022 Aug 8];37(2). Available from: http://pjms.org.pk/index.php/pjms/article/view/2999. doi: 10.12669/pjms.37.2.2999 33679945 PMC7931310

[37] Schimschal SE, Visentin D, Kornhaber R, Cleary M. Grit: A Concept Analysis. Issues Ment Health Nurs. 2021 May 4;42(5):495–505. doi: 10.1080/01612840.2020.1814913 32915678

[38] Doolittle BR. Association of Burnout with Emotional Coping Strategies, Friendship, and Institutional Support Among Internal Medicine Physicians. J Clin Psychol Med Settings. 2021 Jun;28(2):361–7. doi: 10.1007/s10880-020-09724-6 32415546 PMC7225246

[39] Cortez AR, Winer LK, Kassam AF, Hanseman DJ, Kuethe JW, Sussman JJ, et al. Exploring the relationship between burnout and grit during general surgery residency: A longitudinal, single-institution analysis. Am J Surg. 2020 Feb;219(2):322–7. doi: 10.1016/j.amjsurg.2019.09.041 31623881

[40] Cheema SA, Sajid A, Hassan A. The Association of Grit and Burnout among Gynecological Post-Graduate Residents: A Cross-Sectional Study. Ann King Edw Med Univ. 2020;26(3):462–7.

[41] Walker A, Hines J, Brecknell J. Survival of the Grittiest? Consultant Surgeons Are Significantly Grittier Than Their Junior Trainees. J Surg Educ. 2016 Jul;73(4):730–4. doi: 10.1016/j.jsurg.2016.01.012 27025568

[42] Salles A, Cohen GL, Mueller CM. The relationship between grit and resident well-being. Am J Surg. 2014 Feb;207(2):251–4. doi: 10.1016/j.amjsurg.2013.09.006 24238604

[43] Dam A, Perera T, Jones M, Haughy M, Gaeta T. The Relationship Between Grit, Burnout, and Well-being in Emergency Medicine Residents. Cico SJ, editor. AEM Educ Train. 2019 Jan;3(1):14–9. doi: 10.1002/aet2.10311 30680343 PMC6339541

[44] Sirois FM, Owens J. Factors Associated With Psychological Distress in Health-Care Workers During an Infectious Disease Outbreak: A Rapid Systematic Review of the Evidence. Front Psychiatry. 2021 Jan 28;11:589545. doi: 10.3389/fpsyt.2020.589545 33584364 PMC7876062

[45] Huffman EM, Athanasiadis DI, Anton NE, Haskett LA, Doster DL, Stefanidis D, et al. How resilient is your team? Exploring healthcare providers’ well-being during the COVID-19 pandemic. Am J Surg. 2021 Feb;221(2):277–84. doi: 10.1016/j.amjsurg.2020.09.005 32994041 PMC7486626

[46] Bong CL, Brasher C, Chikumba E, McDougall R, Mellin-Olsen J, Enright A. The COVID-19 Pandemic: Effects on Low- and Middle-Income Countries. Anesth Analg. 2020 Jul;131(1):86–92. doi: 10.1213/ANE.0000000000004846 32243287 PMC7173081

[47] Hickman RL, Pignatiello GA, Tahir S. Evaluation of the Decisional Fatigue Scale Among Surrogate Decision Makers of the Critically Ill. West J Nurs Res. 2018 Feb;40(2):191–208. doi: 10.1177/0193945917723828 28805132 PMC5750078

[48] R Core Team. R: A Language and Environment for Statistical Computing. Vienna: R Foundation for Statistical Computing; 2020.

[49] Agresti A, Coull BA. The analysis of contingency tables under inequality constraints. J Stat Plan Inference. 2002 Sep;107(1–2):45–73.

[50] McNeish D, Stapleton LM, Silverman RD. On the unnecessary ubiquity of hierarchical linear modeling. Psychol Methods. 2017;22(1):114–40. doi: 10.1037/met0000078 27149401

[51] Muth C, Bales KL, Hinde K, Maninger N, Mendoza SP, Ferrer E. Alternative Models for Small Samples in Psychological Research: Applying Linear Mixed Effects Models and Generalized Estimating Equations to Repeated Measures Data. Educ Psychol Meas. 2016 Feb;76(1):64–87. doi: 10.1177/0013164415580432 29795857 PMC5965574

[52] Johns Hopkins CSSE. Coronavirus COVID-19 Global Cases by John Hopkins [Internet]. Center for Systems Science and Engineering. 2020 [cited 2021 Dec 1]. Available from: https://systems.jhu.edu/research/public-health/ncov/.

[53] Secretaría Distrital de Salud. Observatorio de Salud de Bogotá-SaluData [Internet]. SaluData. 2020 [cited 2021 Dec 1]. Available from: https://saludata.saludcapital.gov.co/osb.

[54] Leng M, Wei L, Shi X, Cao G, Wei Y, Xu H, et al. Mental distress and influencing factors in nurses caring for patients with COVID-19. Nurs Crit Care. 2021 Mar;26(2):94–101. doi: 10.1111/nicc.12528 33448567

[55] Danziger S, Levav J, Avnaim-Pesso L. Extraneous factors in judicial decisions. Proc Natl Acad Sci. 2011;108(17):6889–6892. doi: 10.1073/pnas.1018033108 21482790 PMC3084045

