# Supplementary material for: Compassion and decision fatigue among healthcare workers during COVID-19 pandemic in a Colombian sample
Source: PLoS One. 2023 Mar 24;18(3):e0282949. doi: 10.1371/journal.pone.0282949 (PMC10038311; doi:10.1371/journal.pone.0282949)
Supplement: S1 Fig — (DOCX) [file pone.0282949.s001.docx]

**S3. Interaction results**

**
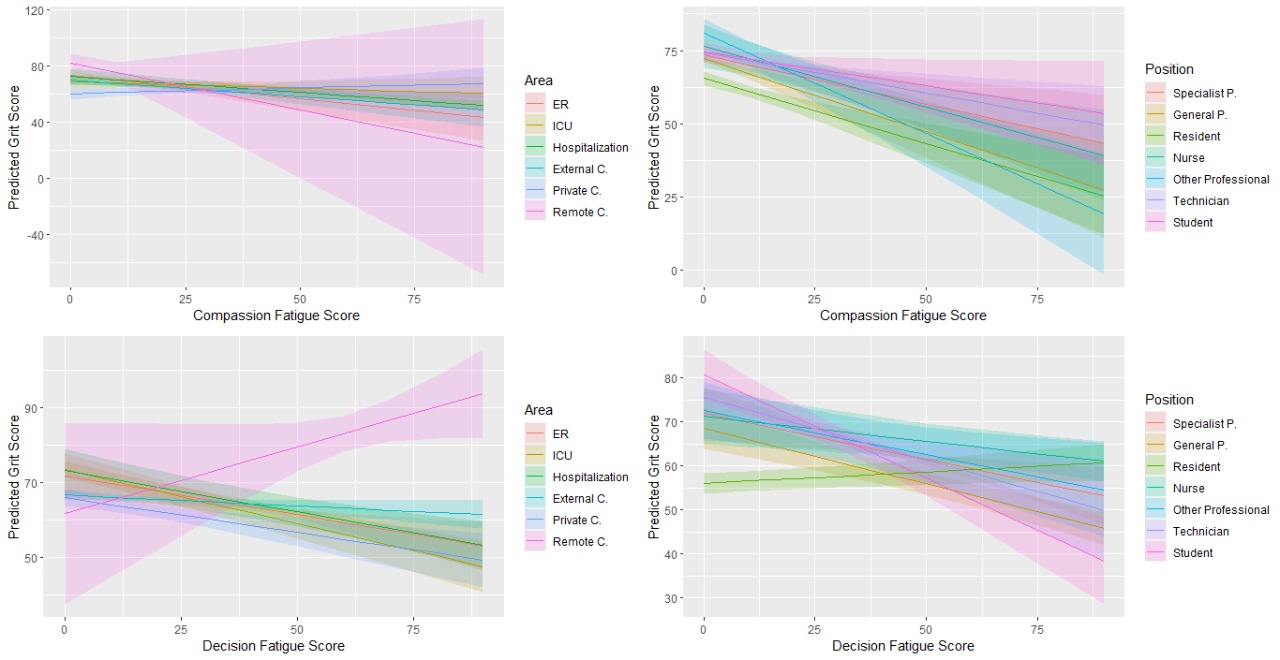
**

**Fig 1.** (A) Interaction between Compassion fatigue and Service area to predict Grit (B) Interaction between Compassion fatigue and Position to predict Grit (C) Interaction between Decision fatigue and Service area to predict Grit (D) Interaction between Decision fatigue and Position to predict Grit. Lines show means and shaded areas confidence intervals.


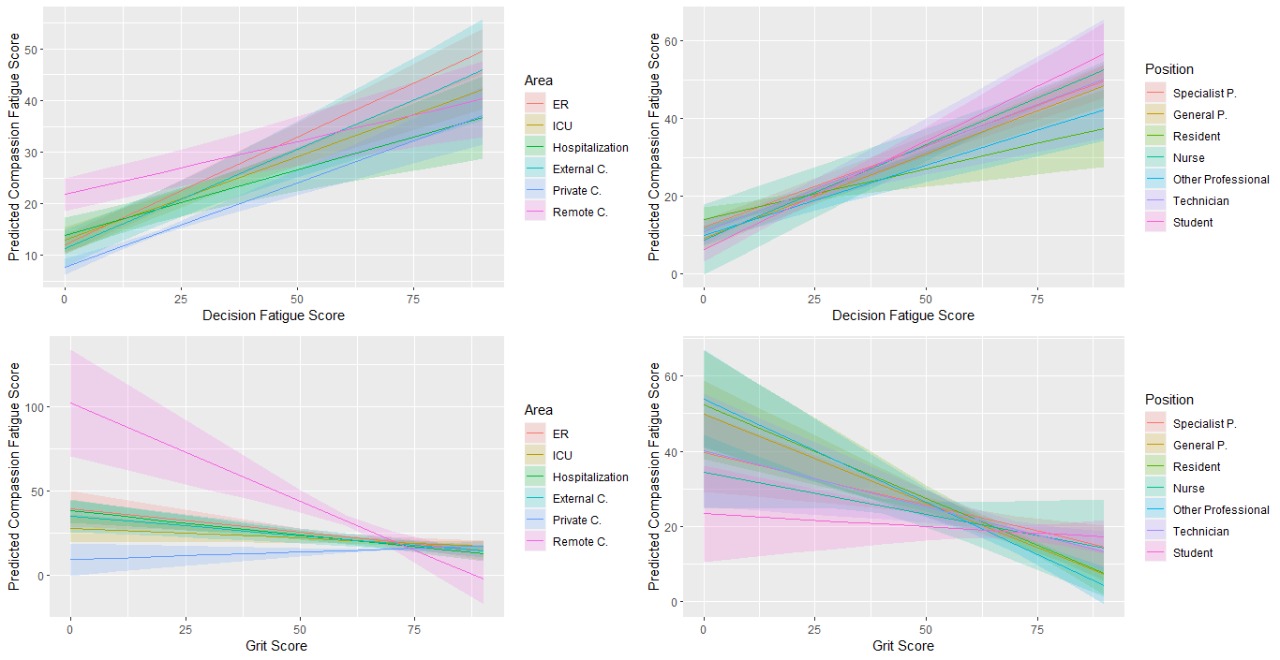


**Fig 2.** (A) Interaction between Decision fatigue and Service area to predict Compassion fatigue (B) Interaction between Decision fatigue and Position to predict Compassion fatigue (C) Interaction between Grit and Service area to predict Compassion fatigue (D) Interaction between Grit and Position to predict Compassion fatigue. Lines show means and shaded areas confidence intervals.


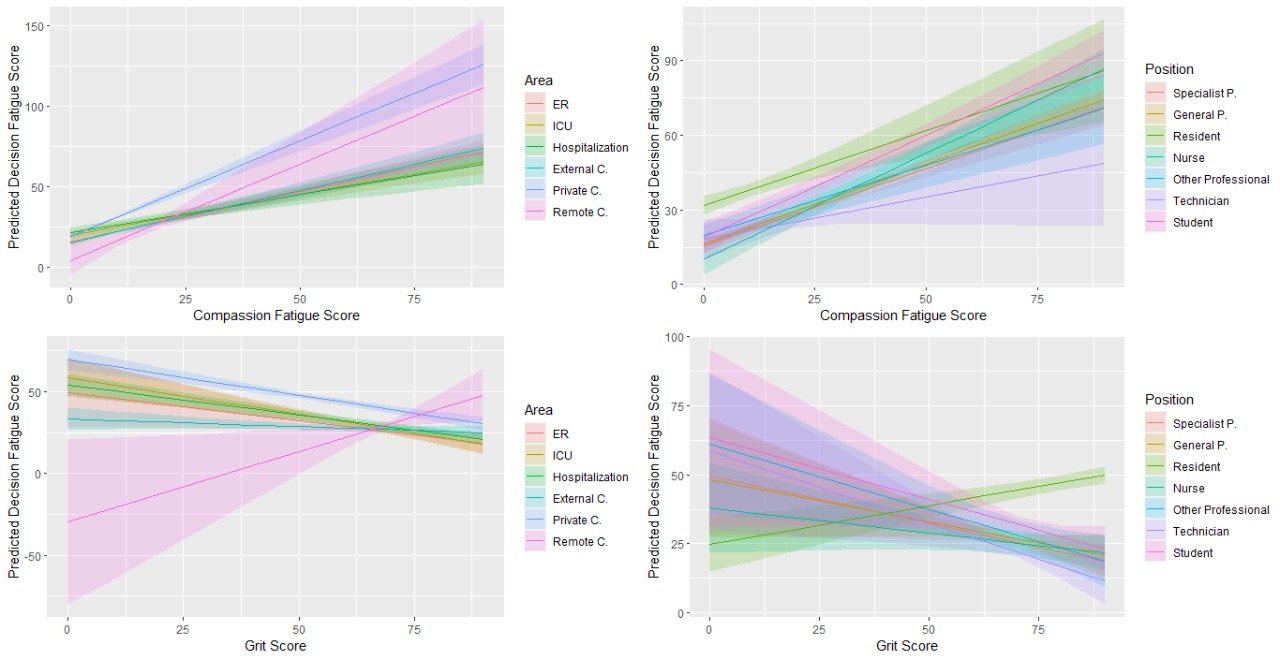


**Fig 3.** (A) Interaction between Compassion fatigue and Service area to predict Decision fatigue (B) Interaction between Compassion fatigue and Position to predict Decision fatigue (C) Interaction between Grit and Service area to predict Decision fatigue (D) Interaction between Grit and Position to predict Decision fatigue. Lines show means and shaded areas confidence intervals.
