# Supplementary material for: Compassion and decision fatigue among healthcare workers during COVID-19 pandemic in a Colombian sample
Source: PLoS One. 2023 Mar 24;18(3):e0282949. doi: 10.1371/journal.pone.0282949 (PMC10038311; doi:10.1371/journal.pone.0282949)

**S2. Comparisons of the results using the full data set, the data set without repeated IP Addresses and the data set without outliers.**

Means

full data

| Peak | Cases | Grit | Decision_Fatigue | Compassion_Fatigue |
| --- | --- | --- | --- | --- |
| Peak | 105 | 3.33 (0.74) - [1.16666666666667--4.5] | 0.87 (0.77) - [0--3] | 3.33 (1.82) - [1--9.18181818181818] |
| Post-Peak | 278 | 3.5 (0.7) - [0.833333333333333--4.5] | 0.64 (0.69) - [0--3] | 2.49 (1.66) - [1--10] |
| Pre-Peak | 473 | 3.32 (0.68) - [1.33333333333333--4.5] | 0.8 (0.73) - [0--3] | 2.97 (1.78) - [1--8.63636363636364] |
| Total | 856 | - | - | - |

Means IP Addresses

| Peak | Cases | Grit | Decision_Fatigue | Compassion_Fatigue |
| --- | --- | --- | --- | --- |
| Peak | 92 | 3.39 (0.74) - [1.16666666666667--4.5] | 0.87 (0.8) - [0--3] | 3.37 (1.88) - [1--9.18181818181818] |
| Post-Peak | 271 | 3.49 (0.7) - [0.833333333333333--4.5] | 0.62 (0.67) - [0--3] | 2.45 (1.59) - [1--10] |
| Pre-Peak | 459 | 3.32 (0.69) - [1.33333333333333--4.5] | 0.8 (0.73) - [0--3] | 2.97 (1.76) - [1--8.63636363636364] |
| Total | 822 | - | - | - |

Means Outliers

| Peak | Cases | Grit | Decision_Fatigue | Compassion_Fatigue |
| --- | --- | --- | --- | --- |
| Peak | 101 | 3.34 (0.7) - [1.5--4.5] | 0.81 (0.71) - [0--2.71428571428571] | 3.25 (1.7) - [1--7.54545454545455] |
| Post-Peak | 272 | 3.52 (0.67) - [1.5--4.5] | 0.6 (0.62) - [0--2.57142857142857] | 2.4 (1.46) - [1--6.81818181818182] |
| Pre-Peak | 467 | 3.33 (0.68) - [1.33333333333333--4.5] | 0.79 (0.71) - [0--2.85714285714286] | 2.95 (1.74) - [1--8.63636363636364] |
| Total | 840 | - | - | - |

ANOVAs

Grit Full data


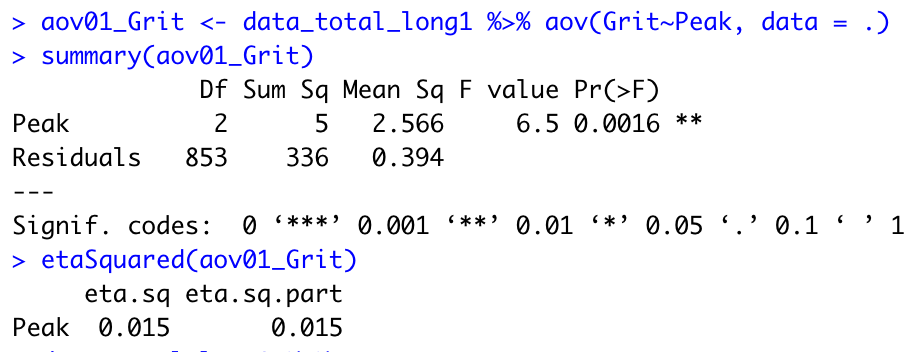

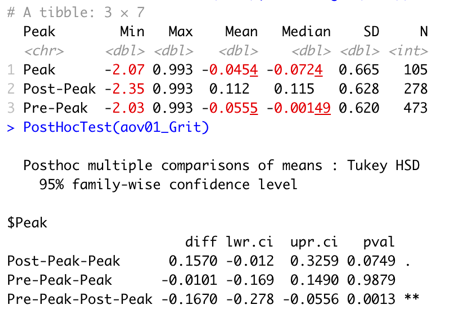


Grit IP Address


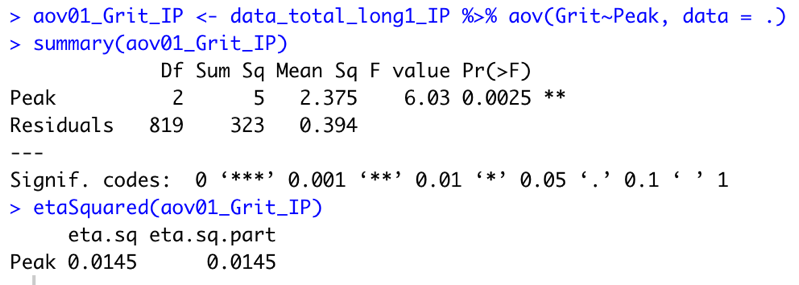

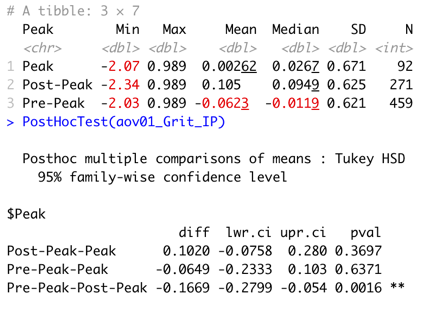


Grit Outliers


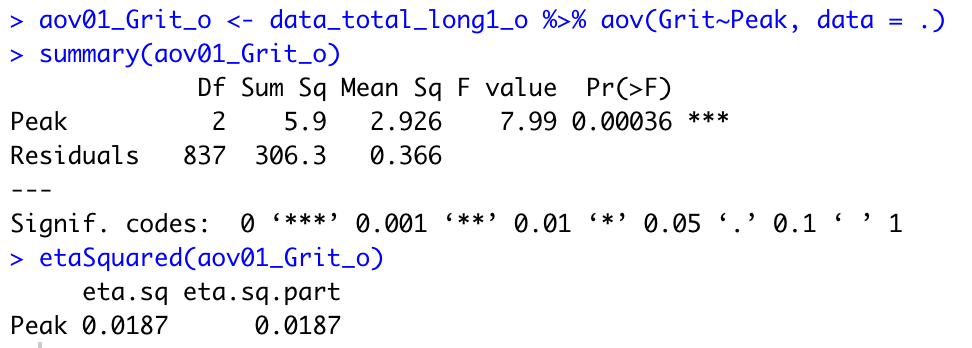

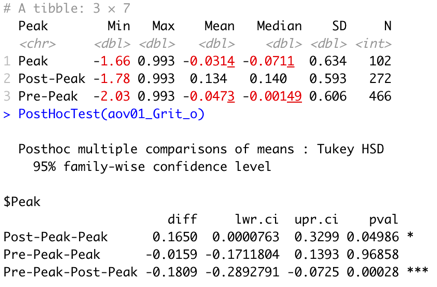


Decision fatigue full data


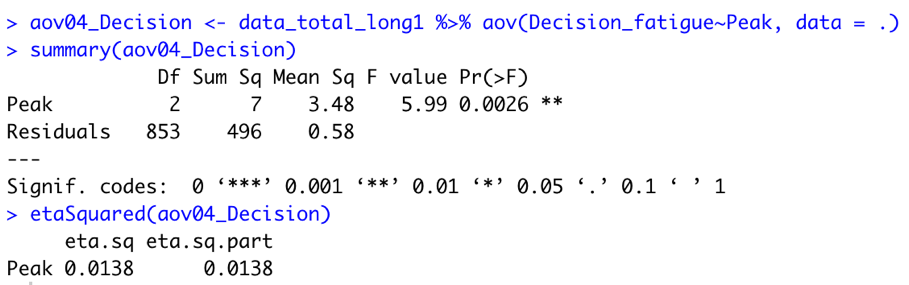

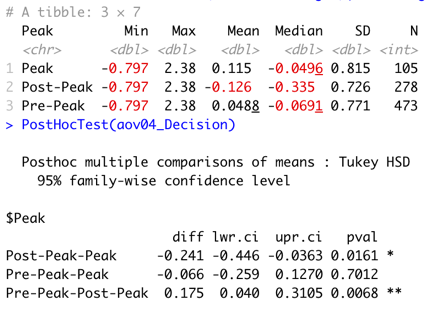


Decision fatigue IP Addresses


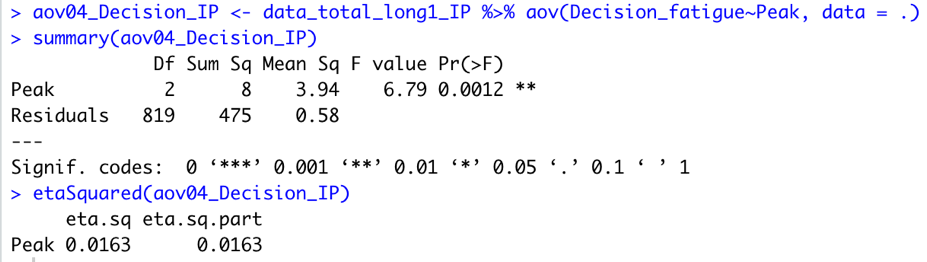

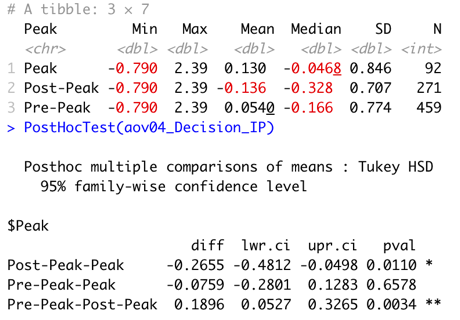


Decision fatigue outliers


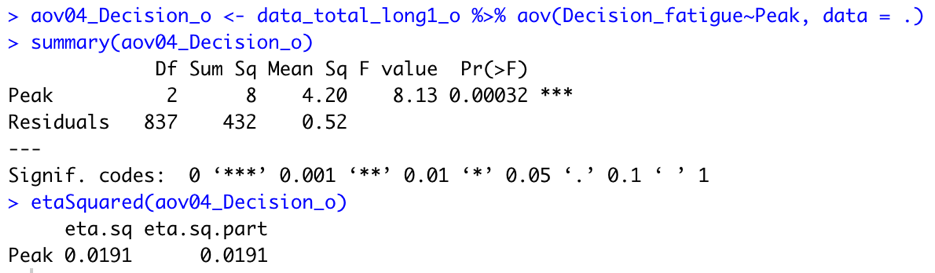

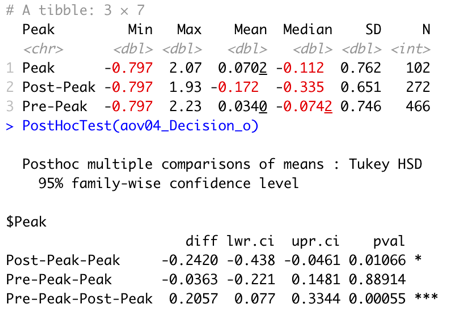


Compassion fatigue full data


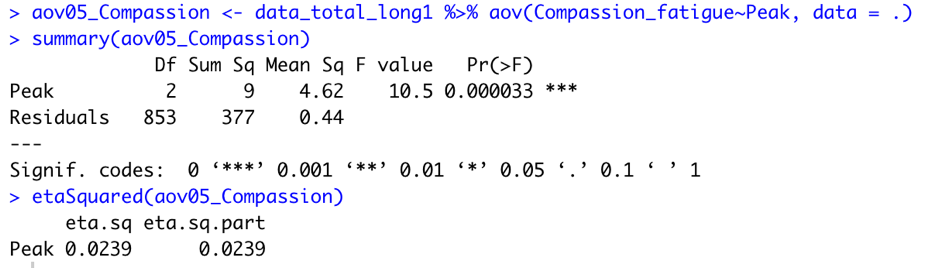

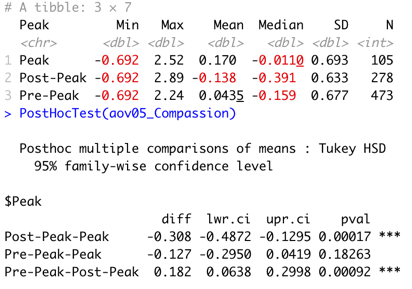


Compassion fatigue IP Addresses


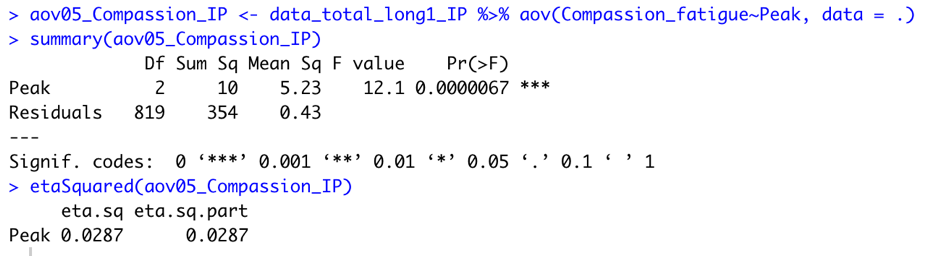

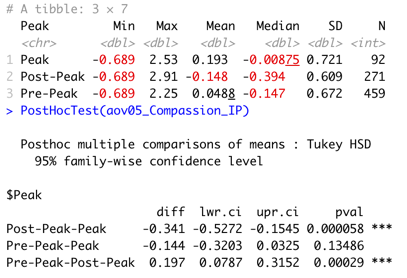


Compassion fatigue outliers


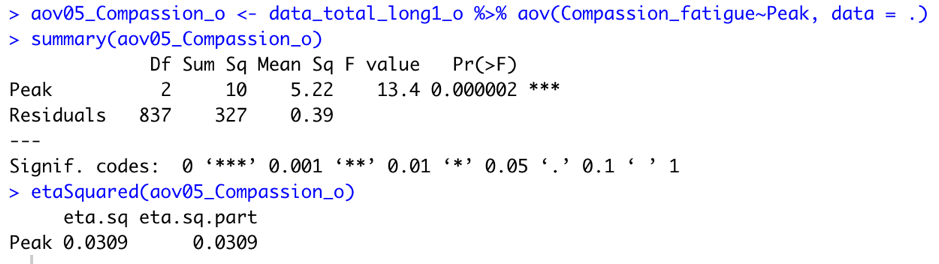

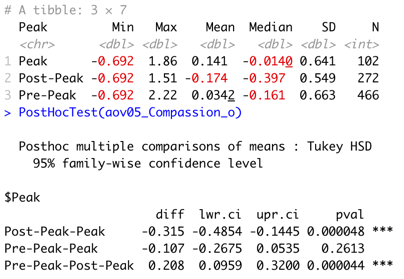


ICC

Grit ICC full data – IP Address – Outliers


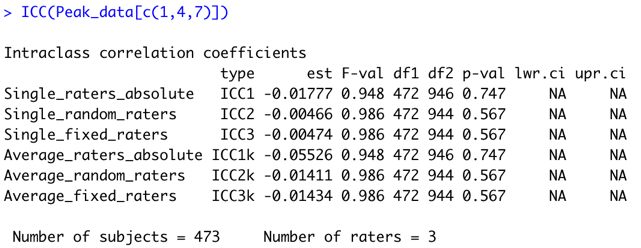

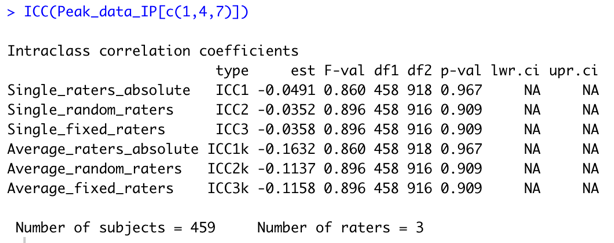

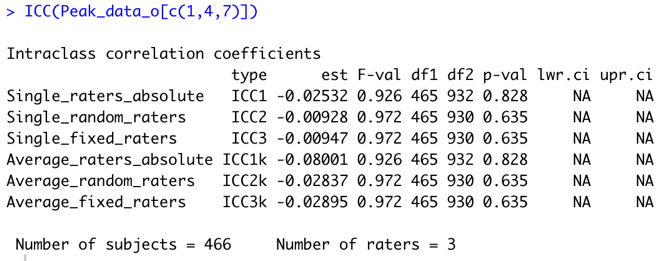


Compassion fatigue ICC full data – IP Address – Outliers


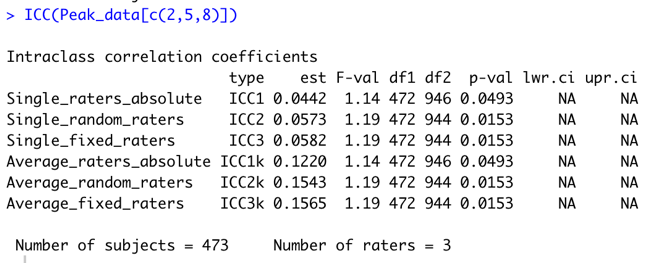

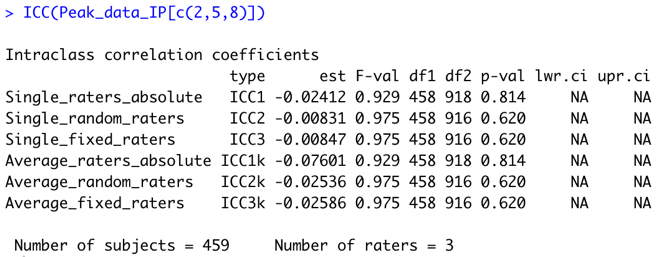

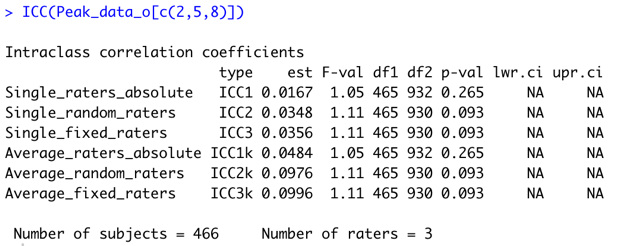


Decision fatigue ICC full data – IP Address – Outliers


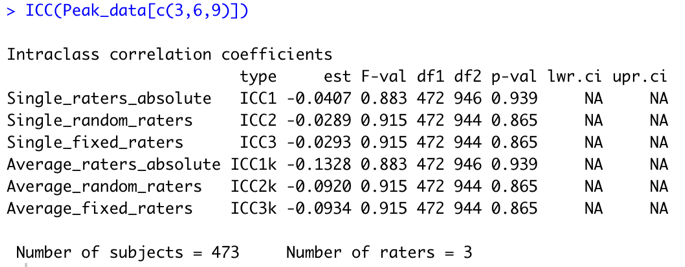

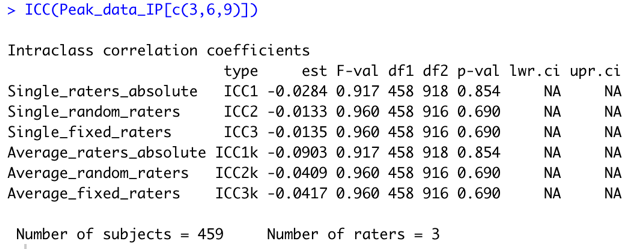

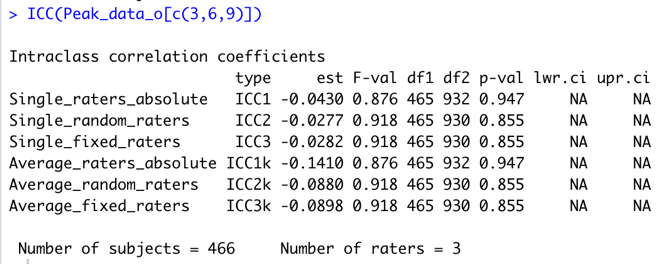


Levene Grit ICC full data – IP Address – Outliers


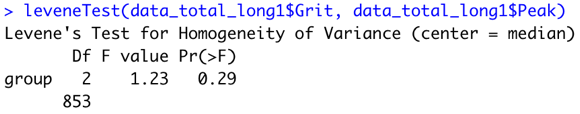

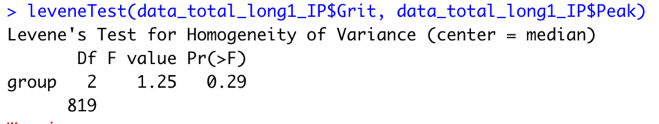

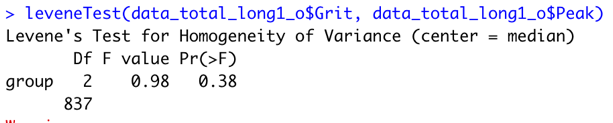


Levene Compassion fatigue ICC full data – IP Address – Outliers


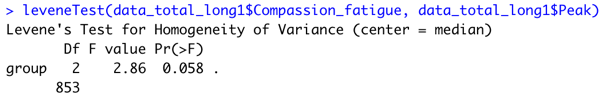

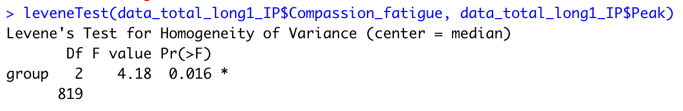

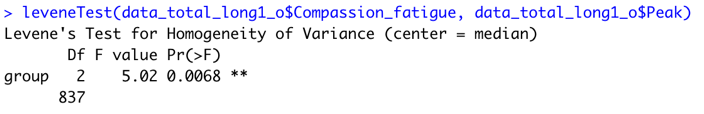


Levene Decision fatigue ICC full data – IP Address – Outliers


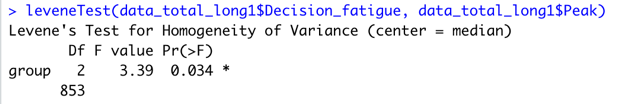

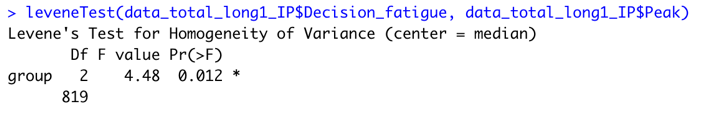

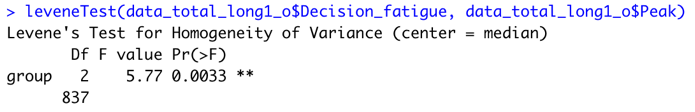


GEELMs

Grit GEELM full data – IP Address – Outliers


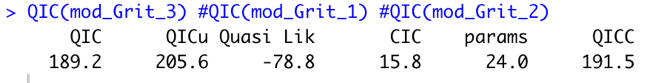

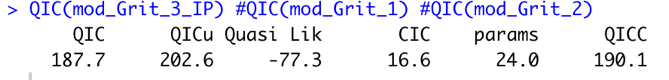

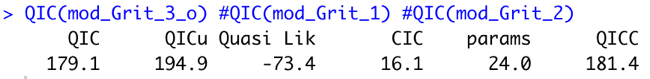


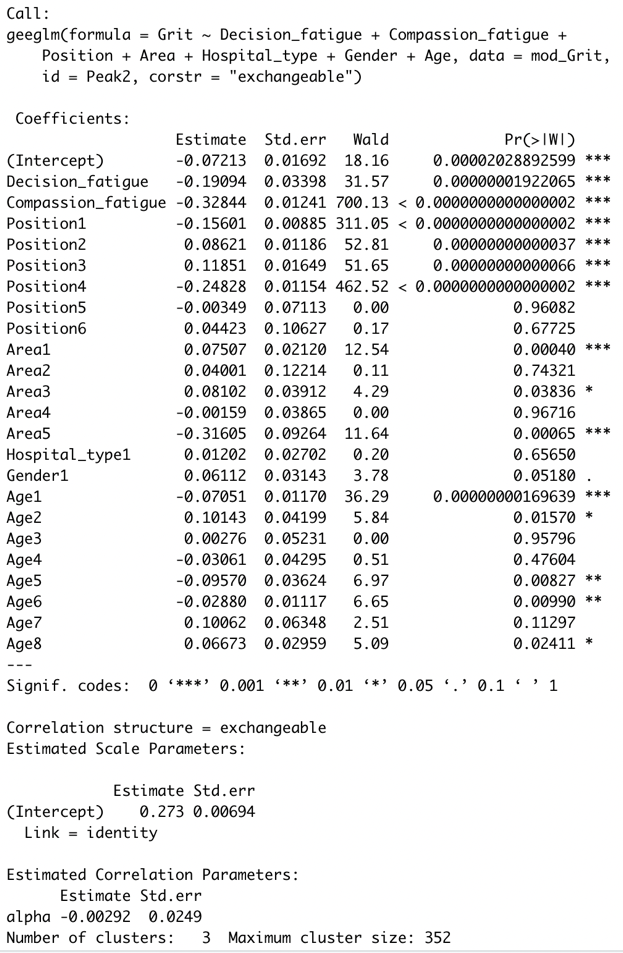

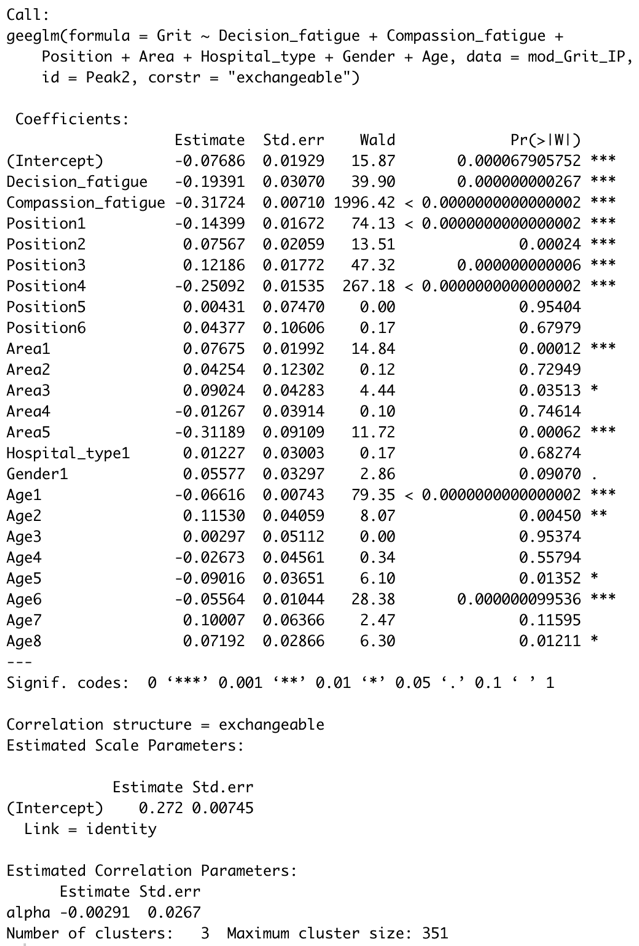

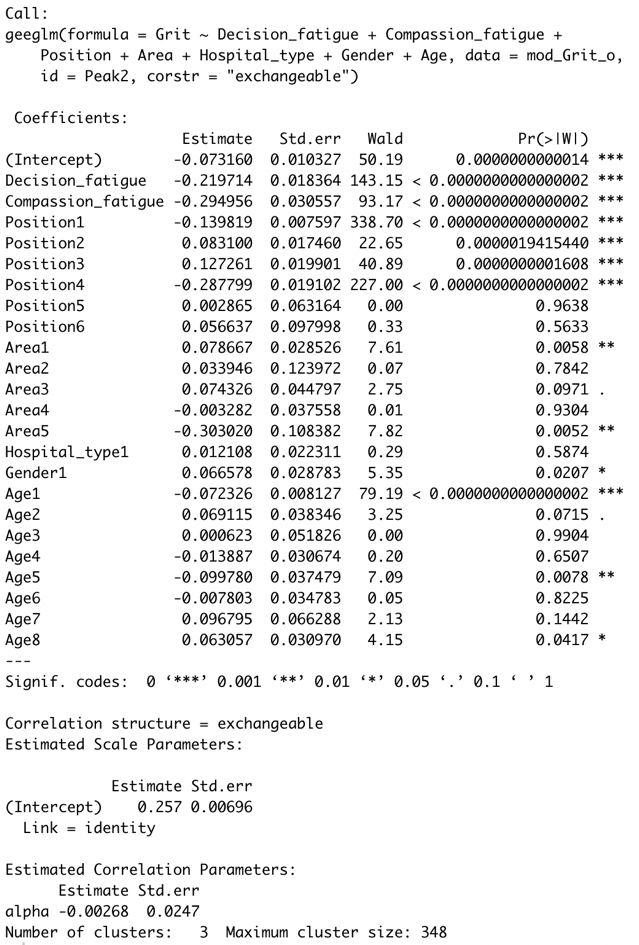


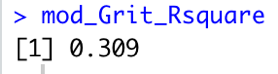

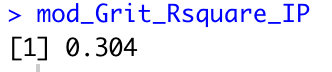

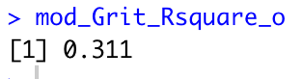


Grit GEELM with FDR correction full data – IP Address – Outliers


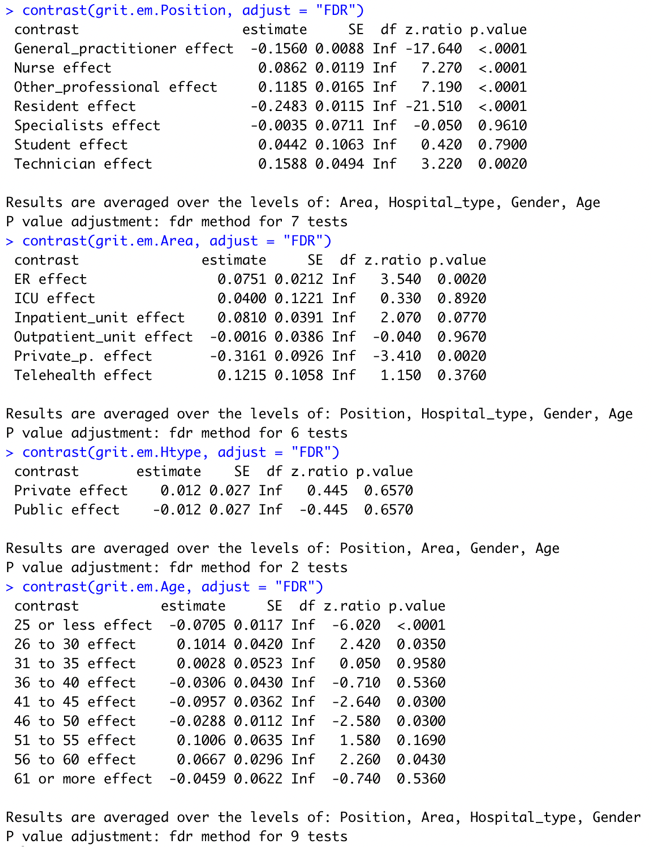

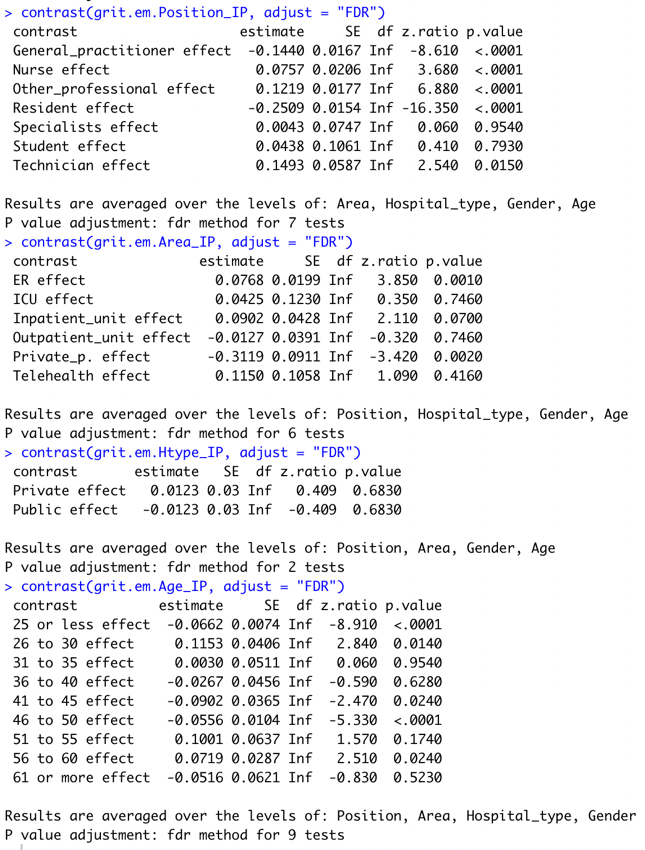

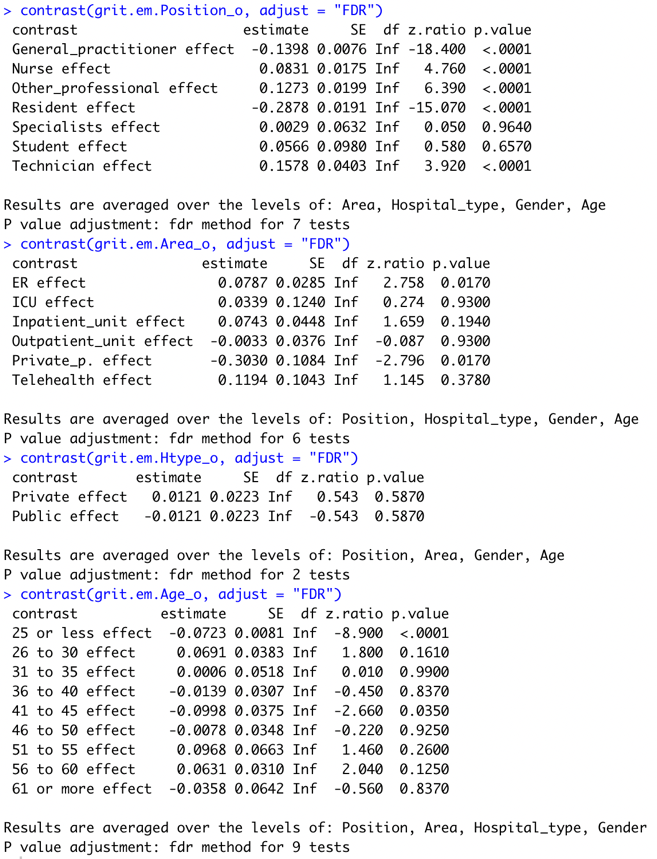


GEELM Decision fatigue full data – IP Address – Outliers


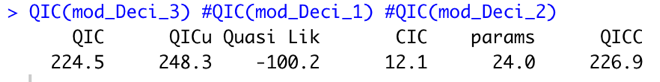

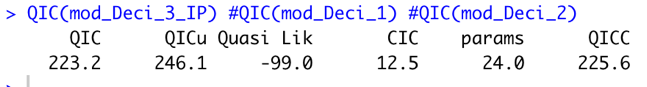

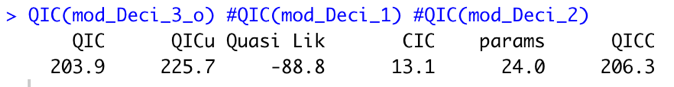


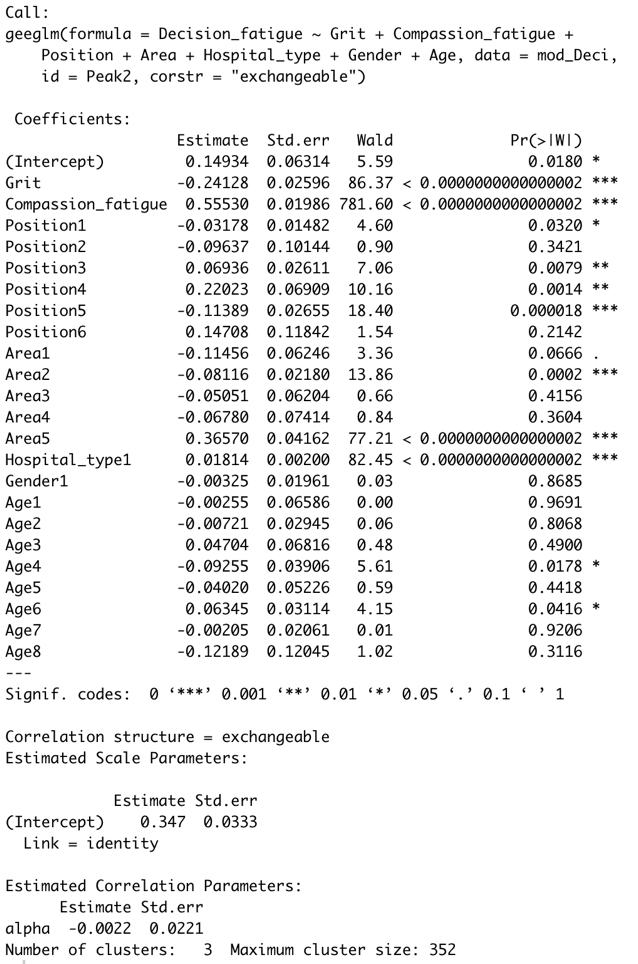

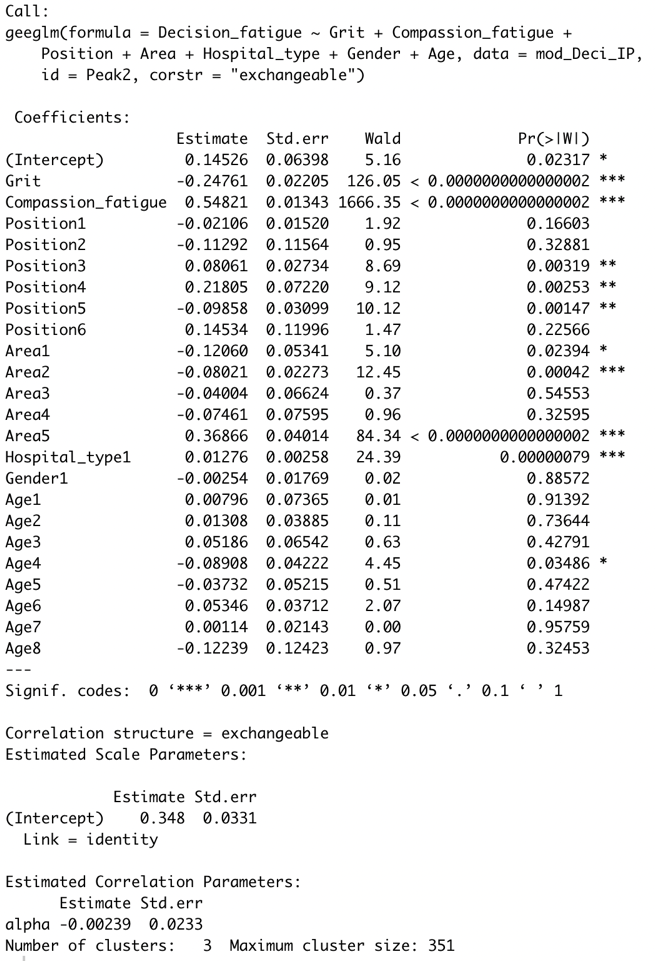

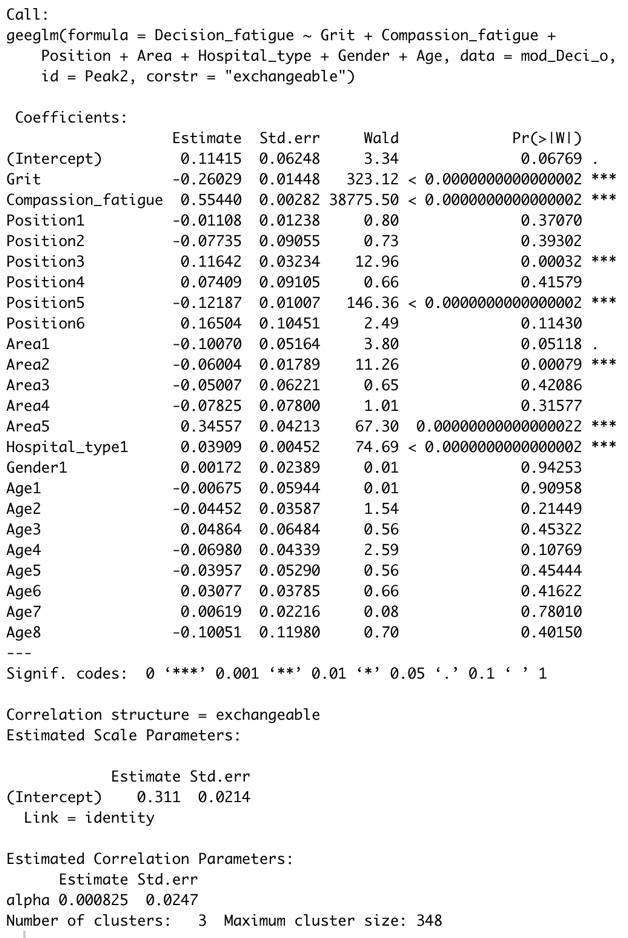


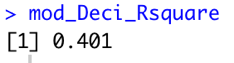

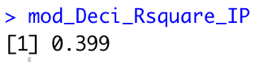

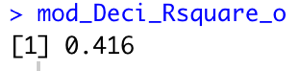


Decision fatigue GEELM with FDR correction full data – IP Address – Outliers


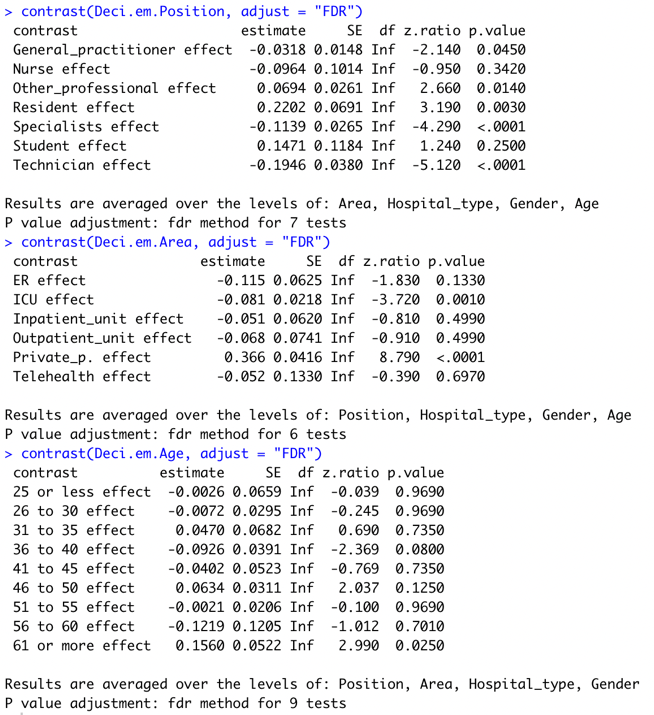

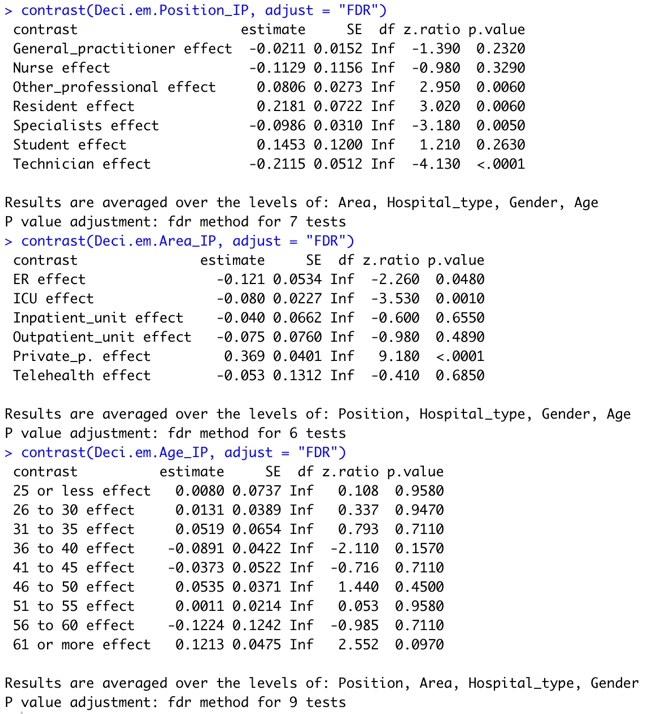

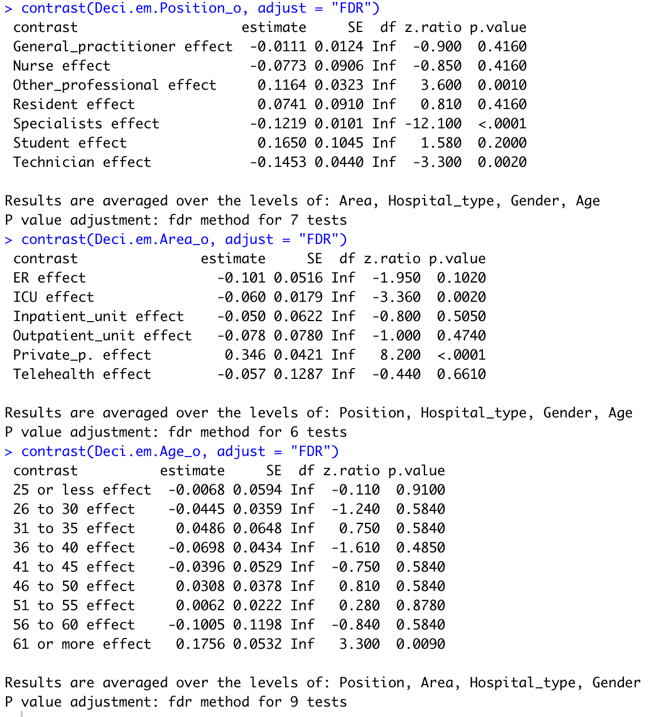


GEELM Compassion fatigue full data – IP Address – Outliers


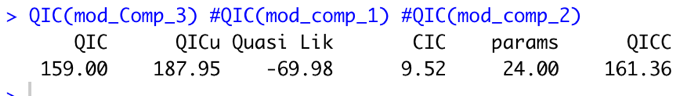

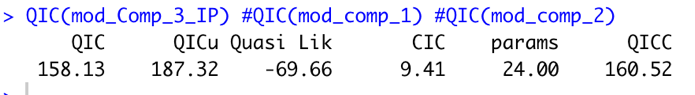

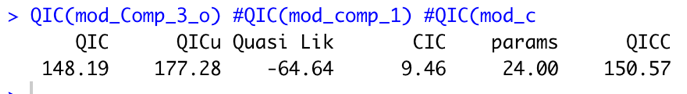


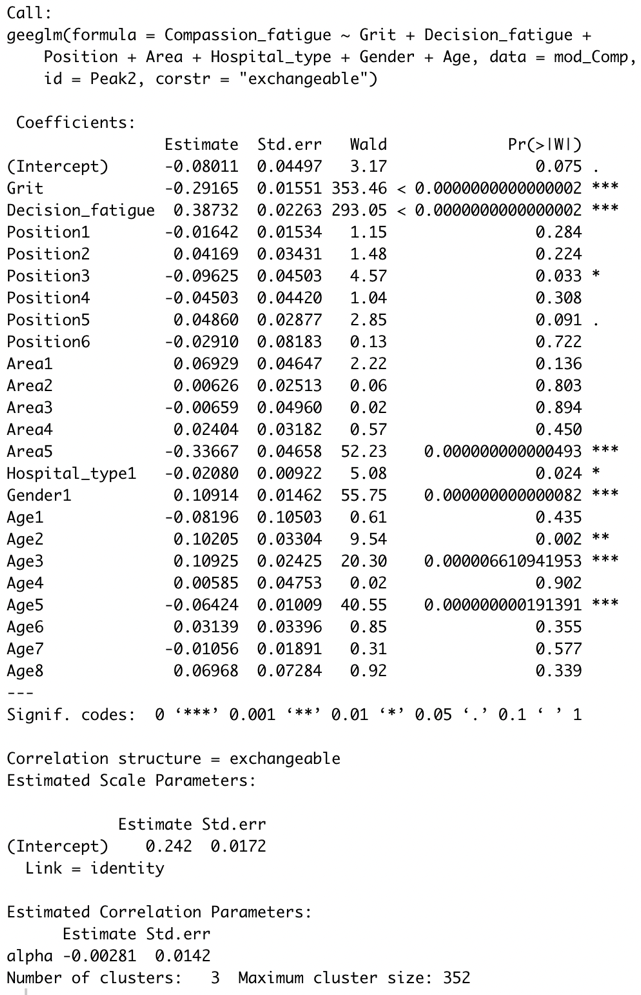

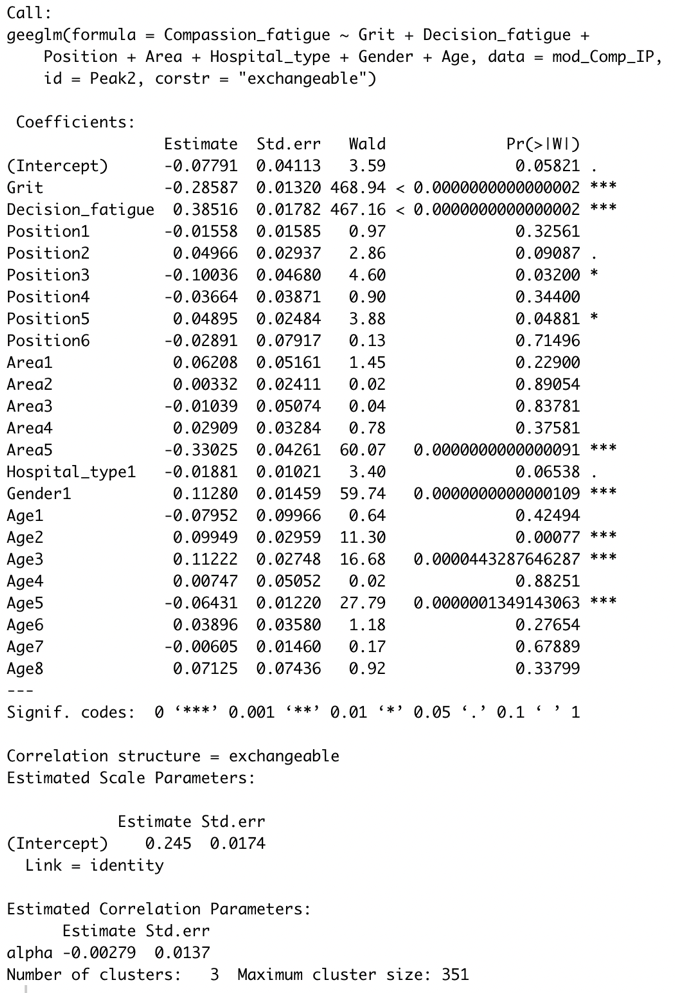

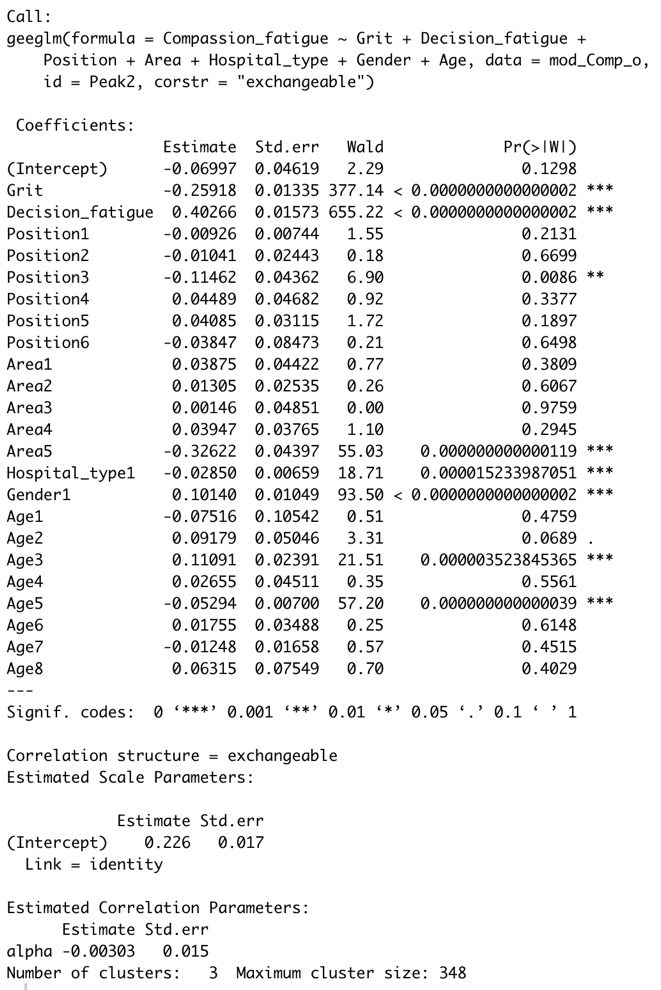


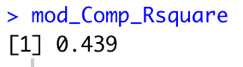

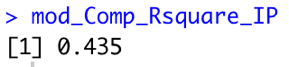

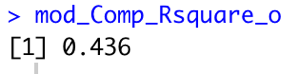


Compassion fatigue GEELM with FDR correction full data – IP Address – Outliers


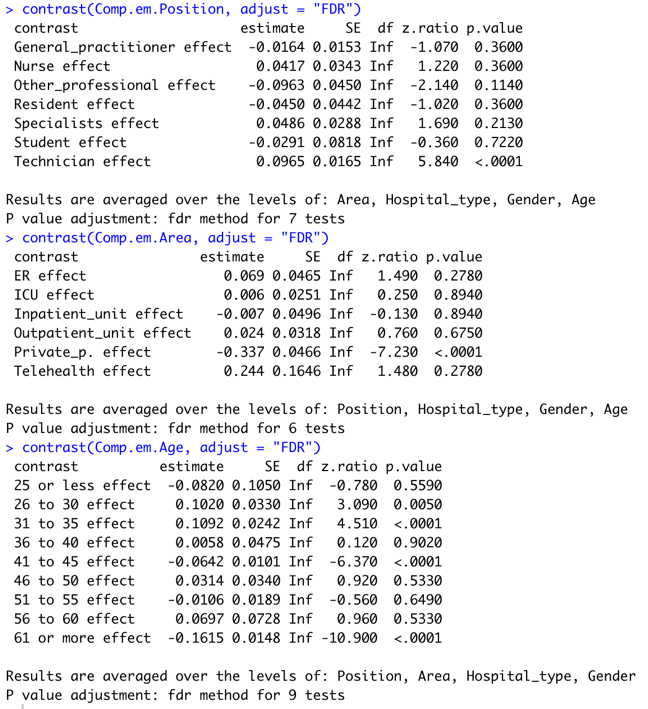

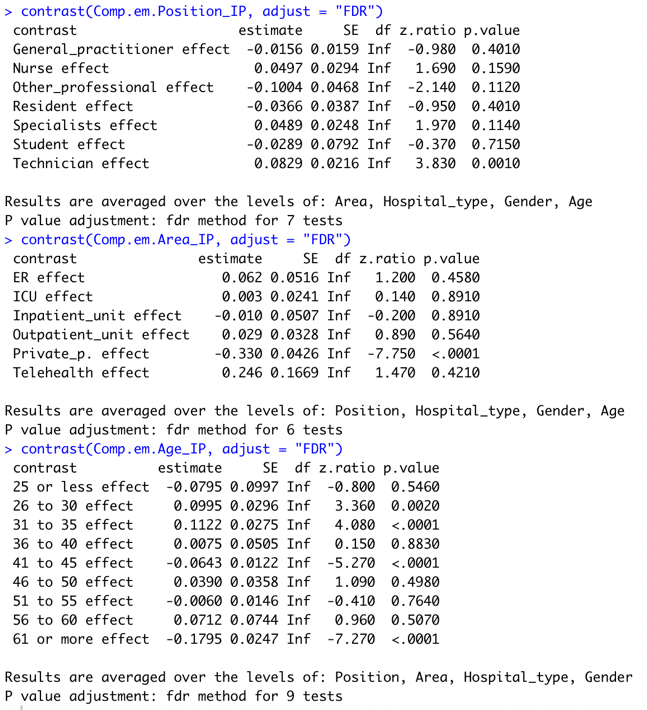

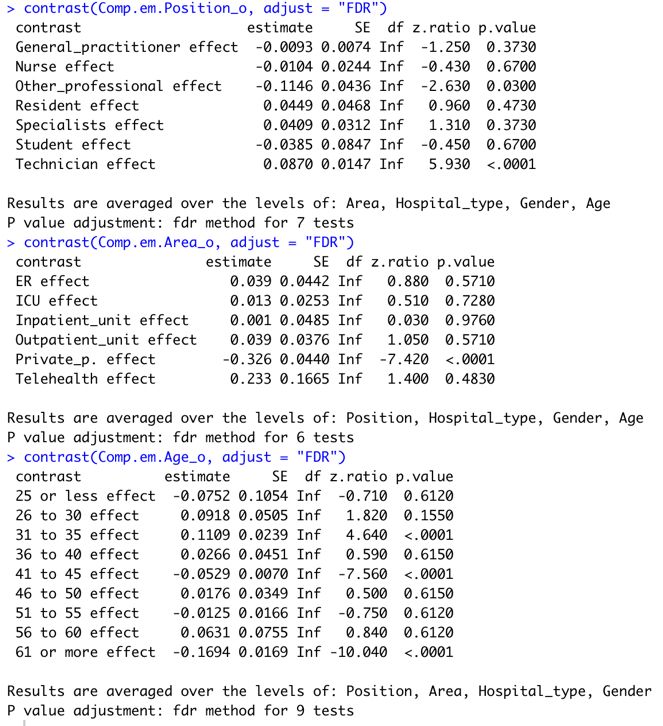

Supplement: S1 Table — (DOCX) [file pone.0282949.s002.docx]
