# Supplementary material for: Compassion and decision fatigue among healthcare workers during COVID-19 pandemic in a Colombian sample
Source: PLoS One. 2023 Mar 24;18(3):e0282949. doi: 10.1371/journal.pone.0282949 (PMC10038311; doi:10.1371/journal.pone.0282949)
Supplement: S1 File — (DOCX) [file pone.0282949.s003.docx]

**S1.** **Full instruments and translations**

**1. Grit**

**Original.** *Short Grit Scale (Grit-S)* . (Duckworth & Quinn, 2009)

The instrument is rated using a 5-point Likert-type scale ranging from 1 (*Not at all like me*) to 5 (*Very* *much like me*). According to the author´s validation, the instrument has good internal consistency with alphas ranging from .73 to .83. Both factors (consistency of interest and perseverance of effort) showed adequate internal consistency and were strongly intercorrelated, *r* = .59, *p <*.001.

Factor 1. Consistency of interest

1. I often set a goal but later choose to pursue a different one.

2. I have been obsessed with a certain idea or project for a short time but later lost interest.

3. New ideas and projects sometimes distract me from previous ones.

Factor 2. Perseverance of effort

4. I finish whatever I begin.

5. Setbacks don’t discourage me. I don’t give up easily.

6. I am diligent. I never give up.

Removed items: *I am a hard worker* and *I have difficulty maintaining my focus on projects that take more than a few months to complete*

The observed reliability for the 6-item translated instrument that was used in our study sample was α = 0.7 and ω = 0.77.

**Translated:** *Escala corta de determinación (Grit-S)*.

Se utiliza una escala tipo Likert de 5 puntos que va desde 1 (para nada como yo) hasta 5 (muy parecido a mi).

Factor 1. Consistencia en el interés

1. Frecuentemente me pongo una meta, pero después decido perseguir otra diferente.

2. He estado obsesionado con alguna idea o proyecto por un corto tiempo, pero luego he perdido el interés.

3. Las nuevas ideas y proyectos a veces me distraen de los que tenía previamente.

Factor 2. Perseverancia en el esfuerzo

4. Termino todo lo que empiezo.

5. Los obstáculos no me desaniman. No me rindo fácilmente.

6. Soy diligente (hago las cosas con interés, esmero, rapidez y eficacia). Nunca me rindo.

**2. Decision Fatigue**

**Original:** *Decision Fatigue Scale (DFS).* (Hickman et al., 2018)

4-point Likert scale from 0 (strongly disagree) to 3 (strongly agree). Total scores for the DFS are derived by summing the scale items, and the range of total scores is 0 to 30; higher total scores are posited to correlate with the intensity of perceived decision fatigue by the rater. The internal consistency reliability (Cronbach’s alpha) is 0.87 for de 9-item version of the Decision Fatigue Scale.

1. I can’t make a decision because I am too tired and stressed.

2. Making decisions is difficult because I can’t concentrate.

3. It is hard for me to take in information and use it to make decisions.

4. I don’t have enough confidence in myself to make good decisions.

5. It takes too much effort to make decisions.

6. I can’t make up my mind about which option is best.

7. My mood has made it difficult for me to make decisions.

Removed items: *someone else should make decisions for me*, *I have made decisions quickly in order to move on* and *I have made decisions without carefully thinking about them*.

The observed reliability for the 7-items translated instrument that was used in our study sample was α = 0.88 and ω = 0.88.

**Translated:** *Escala de Fatiga de decisión.*

Se utiliza una escala Likert de 4 puntos que va desde 0 (fuertemente en desacuerdo) hasta 3 (fuertemente de acuerdo).

1. No puedo tomar decisiones porque estoy muy cansado y estresado.

2. Tomar decisiones es difícil porque no puedo concentrarme.

3. Es difícil para mí recoger información y usarla para tomar decisiones.

4. No tengo suficiente confianza en mí mismo para tomar decisiones.

5. Tomar decisiones requiere de demasiado esfuerzo.

6. No puedo decidirme sobre cuál es la mejor opción.

7. Mi estado de ánimo ha hecho que sea difícil para mí tomar decisiones.

**3. Compassion Fatigue**

**Original:** Compassion Fatigue Short Scale (CF-Short Scale). (Adams et al, 2006)

Participants are asked to rate the frequency of how often each item applies to themselves on a 10-point Likert scale ranging from 1 (rarely/never) to 10 (very often). According to Adams et al, the Cronbach’s α coefficients of the subscales range from 0.80 to 0.90, demonstrating adequate internal reliability.

Factor 1. Job burnout

1. I have felt depressed because of my work.

2. I have a sense of worthlessness, disillusionment or resentment associated with my work.

3. I have felt trapped by my work.

4. I feel I am unsuccessful at separating work from my personal life.

5. I have felt a sense of hopelessness associated with working with clients/ patients.

6. I feel that I am a ‘failure’ in my work.

7. I have frequently felt weak, tired or rundown because of my work as a caregiver.

Factor 2. Secondary stress

8. I experience troubling dreams similar to those of a client of mine.

9. I have suddenly and involuntarily recalled a frightening experience while working with a client/patient.

10. I am losing sleep over a client’s traumatic experiences.

11. I have had flashbacks connected to my clients.

Removed items: *I have thoughts that I am not succeeding in achieving my life goals* and *I have experienced intrusive thoughts after working with an especially difficult client/patient*.

The observed reliability for the translated 11-item instrument that was used in our study sample was α = 0.88 and ω = 0.89.

**Translated:** *Escala corta de Fatiga por compasión (CF-Short Scale)*

Se utiliza una escala tipo Likert en la que va de 1 (raramente/nunca) a 10 (muy a menudo).

Factor 1. Burnout en el trabajo

1. Me he sentido deprimido debido a mi trabajo.

2. Tengo una sensación de inutilidad, desilusión o resentimiento asociado con mi trabajo.

3. Me he sentido atrapado por mi trabajo.

4. Siento que no he podido separar exitosamente mi trabajo de mi vida personal.

5. He tenido una sensación de desesperanza asociada a trabajar con pacientes.

6. Siento que soy un 'fracaso' en mi trabajo.

7. Frecuentemente me he sentido débil, cansado o en mal estado por mi trabajo como cuidador.

Factor 2. Estrés secundario

8. Experimento sueños inquietantes similares a los que experimenta alguno de mis clientes.

9. He recordado una experiencia aterradora de manera súbita e involuntaria mientras trabajo con un cliente/paciente.

10. Estoy perdiendo el sueño debido a las experiencias traumáticas de mis clientes.

11. He tenido flashbacks relacionados con mis clientes.

**4. Cuestionario de conductas de determinación en trabajadores de la salud.**

Factor 1. Consistencia en el interés

1. Dedico tiempo libre a actualizarme con información sobre el COVID-19.
2. Hablo con frecuencia sobre el COVID-19 y cómo éste afecta mi trabajo en redes sociales (Facebook, Twitter, Whatsapp, etc.).
3. Atender a pacientes con COVID-19 reafirma el valor que tiene mi trabajo para mí.
4. Durante la pandemia del COVID-19 he pensado en la posibilidad de retirarme del trabajo hospitalario.

Factor 2. Perseverancia en el esfuerzo

1. Quiero seguir trabajando durante la pandemia a pesar de la posibilidad de ser discriminado(a) por trabajar en un hospital.
2. Me siento lleno(a) de energía cuando pienso que la pandemia puede acortarse.
3. Me desanima la idea de que los hospitales no tengan suficientes recursos durante la pandemia de COVID-19.

**Translated: Questionnaire of grit behaviors in healthcare workers**

Factor 1. Consistency of interest

1. I use my free time to get updated about COVID-19 information.
2. I frequently talk about COVID-19 and how it affects my job in social media (Facebook, Twitter, Whatsapp, etc.).
3. Caring for COVID-19 patients reaffirms the value of my job.
4. During the COVID-19 pandemic I have thought about the possibility to stop working at hospitals.

Factor 2. Perseverance of effort

1. I want to keep working during the pandemic regardless the possibility of being discriminated for working in a hospital
2. I feel full of energy when I think the pandemic might shorten.
3. It discourages me the idea that the hospitals do not have enough resources during the COVID-19 pandemic.
